# Identifying the most crucial factors associated with depression based on interpretable machine learning: a case study from CHARLS

**DOI:** 10.3389/fpsyg.2024.1392240

**Published:** 2024-07-25

**Authors:** Rulin Li, Xueyan Wang, Lanjun Luo, Youwei Yuan

**Affiliations:** ^1^School of Management, North Sichuan Medical College, Nanchong, China; ^2^Information Centre, Affiliated Hospital of North Sichuan Medical College, Nanchong, China; ^3^School of Management, Huazhong University of Science and Technology, Wuhan, China

**Keywords:** depression, interpretable machine learning, middle-aged and older adults, crucial factors, CHARLS

## Abstract

**Background:**

Depression is one of the most common mental illnesses among middle-aged and older adults in China. It is of great importance to find the crucial factors that lead to depression and to effectively control and reduce the risk of depression. Currently, there are limited methods available to accurately predict the risk of depression and identify the crucial factors that influence it.

**Methods:**

We collected data from 25,586 samples from the harmonized China Health and Retirement Longitudinal Study (CHARLS), and the latest records from 2018 were included in the current cross-sectional analysis. Ninety-three input variables in the survey were considered as potential influential features. Five machine learning (ML) models were utilized, including CatBoost and eXtreme Gradient Boosting (XGBoost), Gradient Boosting decision tree (GBDT), Random Forest (RF), Light Gradient Boosting Machine (LightGBM). The models were compared to the traditional multivariable Linear Regression (LR) model. Simultaneously, SHapley Additive exPlanations (SHAP) were used to identify key influencing factors at the global level and explain individual heterogeneity through instance-level analysis. To explore how different factors are non-linearly associated with the risk of depression, we employed the Accumulated Local Effects (ALE) approach to analyze the identified critical variables while controlling other covariates.

**Results:**

CatBoost outperformed other machine learning models in terms of MAE, MSE, MedAE, and R^2^metrics. The top three crucial factors identified by the SHAP were r4satlife, r4slfmem, and r4shlta, representing life satisfaction, self-reported memory, and health status levels, respectively.

**Conclusion:**

This study demonstrates that the CatBoost model is an appropriate choice for predicting depression among middle-aged and older adults in Harmonized CHARLS. The SHAP and ALE interpretable methods have identified crucial factors and the nonlinear relationship with depression, which require the attention of domain experts.

## 1 Introduction

Population aging is one of the most concerned public health issues. It is predicted that by 2050, China’s elderly population will increase substantially, with more than 400 million people over 65 (Zeng, 2012). Aging leads to a variety of negative health effects, which include an increased risk of depression (Qiu et al., 2020). A report conducted by the World Health Organization showed that depression was ranked as the single largest contributor to non-fatal health loss, and 7% of old adults suffered from depression (World Health Organization, 2017). Moreover, depression can lead to a decline in physical function, lower quality of life, and increased health costs, which will seriously affect the physical and psychiatric health of middle-aged and older adults. In this context, predicting the risk of depression and analyzing the critical predictors of risk formation is crucial for disease control and improving quality of life.

At present, there have been many empirical methods focusing on factors related to depression in middle-aged and older adults (Yunming et al., 2012). Typically, Luo et al. (2023) used binary logistic regression to examine the correlation between dietary diversity, exercise, and depressive symptoms. They showed that a score on the Dietary Diversity Scale and qualified physical activity were protective factors against depressive symptoms among middle-aged women. Jung et al. (2015) conducted multivariable logistic regression and calculated odds ratios to assess potential interactions between depression prevalence and the ages of menarche and menopause. The results found that the odds ratio of depression decreased with increasing age of menopause and duration of reproductive years. However, the above studies have mainly focused on the relationship between physiological aspects and depression.

In addition, some scholars have focused on the relationship between family factors and depression. McMunn et al. (2021) examined the relationship between work-family and middle-aged psychological distress by multivariable logistic regression. They found that middle-aged people with weaker long-term employment relationships had poorer mental health and well-being. Giannelis et al. (2021) used logistic regression to estimate the relationship between family status and depression; the results showed that a higher number of children and lack of cohabitation with a spouse or partner were associated with a greater likelihood of depression. Therefore, since depression has multiple and complex influences, this study comprehensively considered multiple factors such as demographic variables, health status, cognitive status, income, family structure, stress, and life satisfaction.

At the same time, several related studies also focused on the construction of risk prediction models for depression. For example, Zhou et al. (2023) utilized the Kaplan–Meier method and Cox proportional hazards regression model to evaluate the relationship between baseline chronic disease and depression. They found that suffering from different degrees of chronic diseases increased the risk of depression in middle-aged and older adults. Luo et al. (2018) used the Cox proportional hazards model to estimate the relationship between obesity status and depression. They discovered a negative correlation between the relationship between body weight and depression. Kimmel et al. (2000) used Cox proportional hazards regression analysis to predict the mortality hazard associated with depression in chronic hemodialysis outpatients. They found that medical deterioration factors may increase depression. Zhou et al. (2021) used Cox proportional hazards regression models to examine the association between socioeconomic status and the incidence of depression. They found that participants with the highest level of household income had a 20% reduction in risk of depression. Xiang and Wang (2021) used competing risks regression analysis to examine the relationship between childhood adversity and major depression in older adults and found that childhood adversity increased the risk of major depression in later life, especially for those who experienced physical abuse. In the above studies, the risk regression model was able to assess the relationship between depression and risk factors.

However, while parametric models can well illustrate the association between depression and risk factors, many risk-analysis studies have found that these models are still suboptimal when faced with complex relationships and high-dimensional inputs, and machine learning is relatively better (Hao et al., 2019; Luo and Qi, 2021). Recent studies have demonstrated that machine learning methods outperformed traditional linear models regarding nonlinear fitting performance (Zhang et al., 2018). Machine learning is better equipped to identify the relationship between input and output when dealing with multiple inputs or explanatory variables, resulting in more outstanding performance. For example, Lin et al. (2023) classified the trajectories of depressive symptoms via the latent class growth model and growth mixture model. Based on the identified trajectory patterns, three ML classification algorithms, i.e., gradient-enhanced decision tree, support vector machine, and random forest, demonstrated that machine learning could be very robust in predicting depressive symptom onset and developmental trajectory. However, machine learning is often criticized as the ‘black box’ due to its numerous parameters and complex calculation process, which makes the decision-making process not transparent (Tjoa and Guan, 2021).

Therefore, in this study, we propose an idea based on identifying the most critical factors affecting depression using interpretable machine learning. For this purpose, we adopted five machine learning prediction models: Random forest (RF) (Breiman, 2001; Fawagreh et al., 2014), CatBoost (Prokhorenkova et al., 2018; Ibrahim et al., 2020), eXtreme Gradient Boosting (XGBoost) (Chen and Guestrin, 2016), Light Gradient Boosting Machine (LightGBM) (Ke et al., 2017), and Gradient Boosting decision tree (GBDT) (Friedman, 2001; Zhang and Jung, 2019) for depression risk prediction. First, we utilized demographics, cognitive, income, stress, family structure, health status, life satisfaction, and CESD-10 data collected from the China Health and Retirement Longitudinal Study (CHARLS). Then, continuous values of depression in middle-aged and older adults were predicted using demographic variables, health variables, cognitive, income, pressure, family structure, and life satisfaction from CHARLS data. Then, in the baseline experimental study, we randomly divided the dataset into 80% training dataset for model construction and 20% testing dataset for model testing and compared them with the traditional multiple linear regression (LR) model. Based on this, we used SHapley Additive exPlanations (SHAP) (Lundberg and Lee, 2017) to make an in-depth interpretation of the input variables in the prediction model and clarify the importance of each feature and their net impact on different individual cases. We further adopted the Accumulated Local Effects (ALE) (Apley and Zhu, 2020) method to estimate the nonlinear association between crucial variables and predicted depression.

The contribution of this research can be summarized into the following three aspects: (1) We proposed a depression risk prediction method under multiple factors based on the CatBoost model which outperforms traditional parametric model; (2) We proposed interpretable methods based on SHAP and ALE, and explain the decision-making process of the CatBoost model, overcoming the problem of ‘black box’; (3) We identified the most critical risk factors of depression in a hypothesis-free manner based on the interpretable machine learning framework, demonstrating the most crucial position of life satisfaction.

## 2 Materials and methods

### 2.1 Dataset

The data and information used in this study are obtained from the Harmonized CHARLS dataset and Codebook. The development of the Harmonized CHARLS was funded by the National Institute on Aging, and detailed descriptions of the survey design and procedures were reported in the original study documentation (Zhao et al., 2014).

**Figure 1:**
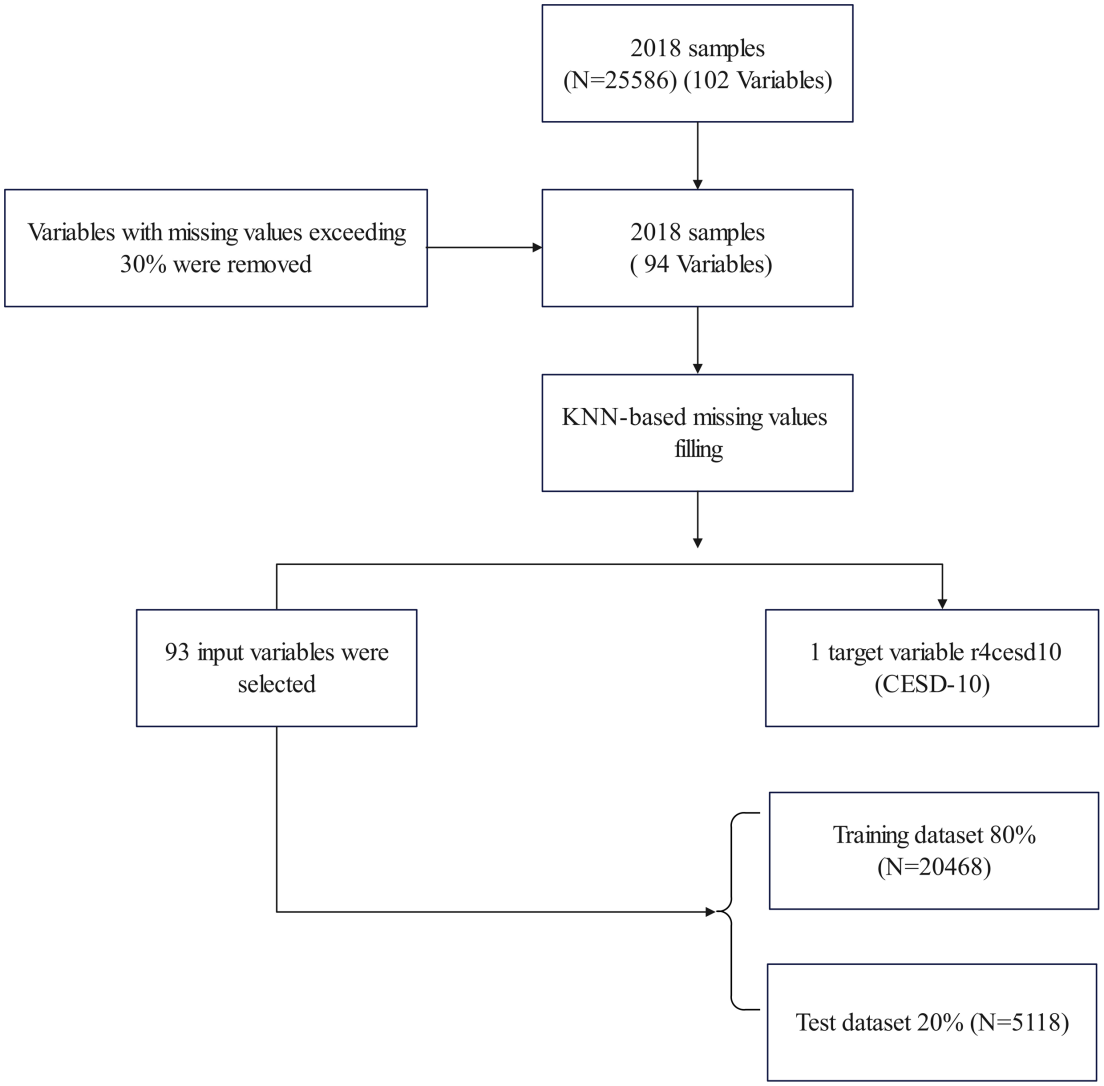
Flow diagram of sample selection.

As shown in Figure 1, in this study, all samples were selected from CHARLS fourth wave ( n=25586 ), and variables with missing values exceeding 30% have been excluded. Finally, ninety-four variables with the proportion of missing values within 30% were used in this study. For these variables, we adopted the KNearest Neighbours (KNN) algorithm (Troyanskaya et al., 2001) to impute the missing values. Based on this, 93 explanatory variables are selected as inputs, and one variable is the prediction target.

### 2.2 Outcome variables and input variables

In this study, the outcome variable is r4cesd10 , which records the continuous value of depression by using the 10-item Center for Epidemiology Studies Depression Scale (CESD-10), which has good reliability and validity among Chinese adults (Chin et al., 2015) and also showed good internal consistency among older respondents (Jiang et al., 2020). CESD-10 focuses on 10 questions about the experience in the past week: feeling depressed, feeling everything they did was effortful, feeling restless sleep, feeling happy, feeling lonely, feeling bothered, feeling they could not get “going,” feeling hopeful for the future, feeling fearful, having trouble in remember what was done. The total CESD-10 score ranged from 0 to 30, with higher scores indicating more severe depressive symptoms. In this study, the CESD-10 depression values of all the respondents were included. A respondent with depression scores of not less than 10 was considered to have depressive symptoms (Chen et al., 2023).

According to the research needs, a total of 93 input variables were selected in the fourth wave of Harmonized CHARLS, including seven categories:

1. Demographic: this category contains eight variables, including rabplace:C (birthplace), raeduc:C (education), r 4mnev (never married), ragender (gender), r4hukou, h4cpl (a couple household), h4rural (urban or rural), r4agey (age).
2. Health status: this category contains 48 variables, including: r4psyche (ever had any emotional, nervous, or psychiatric problems), r4asthmae (ever had asthma), r4memrye (ever had memory-related disease), r4cancre (ever had cancer), r4kidneye (ever had a kidney disease), r4stroke (ever had stroke disease), r4livere (ever had liver disease), r4diabe (ever had diabetes disease), r4lung (ever had lunge disease), r4digeste (ever had a digestive disease), r4hearte (ever had heart disease), r4hibpe (ever had high blood pressure disease), r4dyslipe (ever had dyslipidemia disease), r4arthre (ever had arthritis disease), r4drinkev (ever drinks any alcohol), r4smokev (smoking), r4vgact_c (any vigorous physical activity), r4vgactx_c (the number of days of vigorous activity), r4mdact_c (any moderately physical activity), r4mdactx_c (the number of days of moderate activity), r4ltact_c (any light physical activity), r4ltactx_c (the number of days of light activity), r4mealsa (preparing meals), r4phonea (making phone calls), r4moneya (managing money), r4medsa (taking medications), r4shopa (shopping), r4housewka (cleaning house), r4lowermob (lower body mobility), r4walk1kms (walking 1KM), r4chaira (getting up from a chair), r4mobilsev (7 item mobility), r4dressa (dressing), r4uppermob (3-item summary of any difficulty with upper-body mobility activities), r4stoopa (stooping, kneeling or crouching), r4adlfive (5-item summary of any difficulty with activities of daily living), r4adlab_c (6-item summary), r4adla_c (4-item summary), r4armsa (reaching arms above shoulder level), r 4dlmeas (picking up a coin from the table), r 4lifta (lifting or carrying weights over ten jins), r 4climsa (climbing several flights of stairs without resting), r4toilta (using the toile), r4eata (eating), r4urina (controlling urination and defecation), r4batha (bathing and showering), r4beda (getting in and out of bed), r4shlta (self-reported health).
3. Family structure: this category contains 23 variables, including: h4coresd (any children with them), rameduc_c (mother’s education level), rafeduc_c (father’s education level), h4dchild (total number of deceased children), h4child (number of living children), r4dadliv (father alive), h4kcnt (contact with their children), h4lvnear (live near children), r4livpar (number of living parents), r4dadoccup_c (father’s occupation), r4momliv (the respondent’s mother is alive), h4fcamt (amount of transfers from children/grandchildren), h4tcamt (amount of transfers to children/grandchildren), h4fpamt (amount of transfers from parents/parents-in-law), h4tpamt (amount of transfers to parents/parents-in-law), h4foamt (the number of transfers from others), h4toamt (the number of transfers to others), h4frec (the total amount of transfers received), h4tgiv (the total amount of transfers given), h4ftot (net value of financial transfers), r4decsib (number of deceased siblings), r4livsib (number of living siblings), r4socwk (social activities).
4. Income: r4ipen (private pension).
5. Stress: this category contains 11 variables, including ramomdrug (female guardian had an alcohol and drug), rapadrug (guardians had alcoholism nor had a drug problem), ramwarm_c (female guardian warmth summary mean score), ramomgrela (good relationship with female guardian), ramomeft (female guardian put effort into watching over), ramomatt_c (received female guardian’s love), radaddrug (male guardian had an alcohol and drug), radadgrela (good relationship with male guardian), rafinacom (self-rated family financial situation before age 17), Rahltcom (health condition compared to other children), r4chdeathe (experienced death of own child),
6. Cognitive: r4slfmem (self-reported memory).
7. Life Satisfaction: r4satlife.

The details, including meanings and descriptive analysis of the input and outcome variables, can be found in the Supplementary material.

### 2.3 Predictive model development and evaluation

In this study, compared to the traditional multiple linear regression model, machine learning models of the XGBoost, GBDT, RF, CatBoost, and LightGBM were used to predict the risk of depression and generate six sets of data. Among them, the LR Model is a classic statistical algorithm. XGBoost, LightGBM, and CatBoost are the current most used tree-based algorithms, which can also be classified into the gradient-boosting decision tree algorithm series.

For the hyperparameter setting, Catboost was selected as the primary model for this study due to its excellent performance in healthy prediction-related studies (Zhang et al., 2021), and Optuna (Akiba et al., 2019) was used for optimal parameter search. In detail, for Catboost, the loss:function is set to MAE , the l2:leaf:reg is fixed to 0.0034, the learning:rate is 0.0155, the n:estimators is 22,148, the depth is 7, the min:data:in:leaf is 13. All other model parameters use the default settings from Python libraries: scikit-learn (version 1.2.2), xgboost (version 1.7.5), and lightgbm (version 4.3).

In the regression task, we used four common metrics to evaluate the models’ performances, including mean absolute error (MAE), mean square error (MSE), median absolute error (MedAE), and R-squared. For MAE, MSE, and MedAE, the smaller error, the better the model. In addition, R^2^ measures how well the model explains the total variance of the outcome variable; the closer R^2^ is to 1, the better the model’s fit. The calculations of these four metrics are shown in Equations 1–4.


(1) $$MAE=\frac{1}{n}\overset{n}{\underset{i=1}{\sum }}\left|y_{i}-{\hat{y}}_{i}\right|$$

(2) $$MSE=\frac{1}{n}\overset{n}{\underset{i=1}{\sum }}{\left(y_{i}-{\hat{y}}_{i}\right)}^{2}$$

(3) $$\mathrm{MedAE}=\mathrm{median}\left(\left|y_{1}-{\hat{y}}_{1}\right|,\ldots ,\left|y_{n}-{\hat{y}}_{n}\right|\right)$$

(4) $$R^{2}=1-\frac{\sum_{i=1}^{n}{\left(y_{i}-{\hat{y}}_{i}\right)}^{2}}{\sum_{i=1}^{n}{\left(y_{i}-\bar{y}\right)}^{2}}$$

### 2.4 SHAP and ALE methods

Based on the comparison of model prediction effectiveness, the optimal depression risk prediction model can be selected and used as the basis for SHAP and ALE analysis. We used SHAP to find the most critical influencing factors at the entire dataset level and then explained the individual heterogeneity through local sample analysis. Further, we adopted ALE to manipulate selected variables while controlling other covariates to analyze how different factors are nonlinear and associated with depression risk.

The core idea behind SHAP is to assign a value to each feature for a specific prediction, indicating its contribution to the outcome. This approach is based on game theory’s Shapley values, which aim to fairly distribute the gains or losses among players in a cooperative game. Based on this, SHAP treats the machine learning model as a game where each feature is a player. For each prediction, the Shapley value is calculated for each feature, and the overall explanation is obtained by summing up the values for all features. Benefiting from the ability to explain both in individual samples and cumulatively, SHAP can obtain both global and local interpretability. If the SHAP value is positive, as in the case of the regression continuous outcome prediction task, it indicates that the feature increases the prediction and vice versa. A larger absolute value of SHAP means the feature is more critical to the model.

The ALE method aims to analyze how features influence the model’s prediction on average (Molnar, 2020). Consider clarifying how a change in one feature affects the prediction when other input variables are held constant. ALE first divides the input feature’s values into a grid of bins, and for each bin, ALE gets the model’s predictions using the corresponding input value. Based on this, ALE can calculate the difference between the predicted outcome and the overall mean prediction across all bins, namely, the local effect. Through accumulating the regional effects, ALE finally gets the total pattern of each feature. More details on the calculation of SHAP and ALE can be found in Python libraries: Shap library (version 0.41.0) and Alibi (version 0.9.2) (Klaise et al., 2021).

## 3 Results

### 3.1 Model evaluation and comparison

Table 1 demonstrates the performance of each model under the four regression prediction assessment metrics on the test dataset. The performance of the optimal model under each evaluation metric in the table is bolded, and the suboptimal is underlined. It can be seen that the CatBoost model achieved the best performance with MAE (3.2198), MSE (19.6281), MedAE (2.457), and *R*^2^ (0.3855). The sub-optimal modelsare LightGBM and RF. The values of MAE, MSE, MedAE, and *R*^2^ for the LightGBM model, were 3.3809, 19.8245, 2.6759, and 0.3793. The values of MAE, MSE, MedAE, and *R*^2^ for RF model were, respectively, 3.3231, 20.2088, 2.616, 0.3673. According to the evaluation index of model performance, most machine learning models are better than the traditional multiple linear regression model.

**Table 1:** Model performance for predicting depression.

Model	MAE	MSE	MedAE	*R* ^2^
XGBoost	3.4413	21.3028	2.6485	0.333
GBDT	3.4533	20.1564	2.7687	0.3689
RF	3.3231	20.2088	2.616	0.3673
LightGBM	3.3809	19.8245	2.6759	0.3793
CatBoost	**3.2198**	**19.6281**	**2.457**	**0.3855**
LR	3.5455	20.8503	2.8527	0.3472

Bold value means optimal performance in the corresponding metric.

It is worth noting that, in the baseline experimental study, the outcome variable was also performed KNN-based missing value filling to retain more samples. In the Sect.3.4 robustness test, we further excluded samples with null values of the outcome variable to verify the reliability of the study.

### 3.2 SHAP analysis results

**Figure 2:**
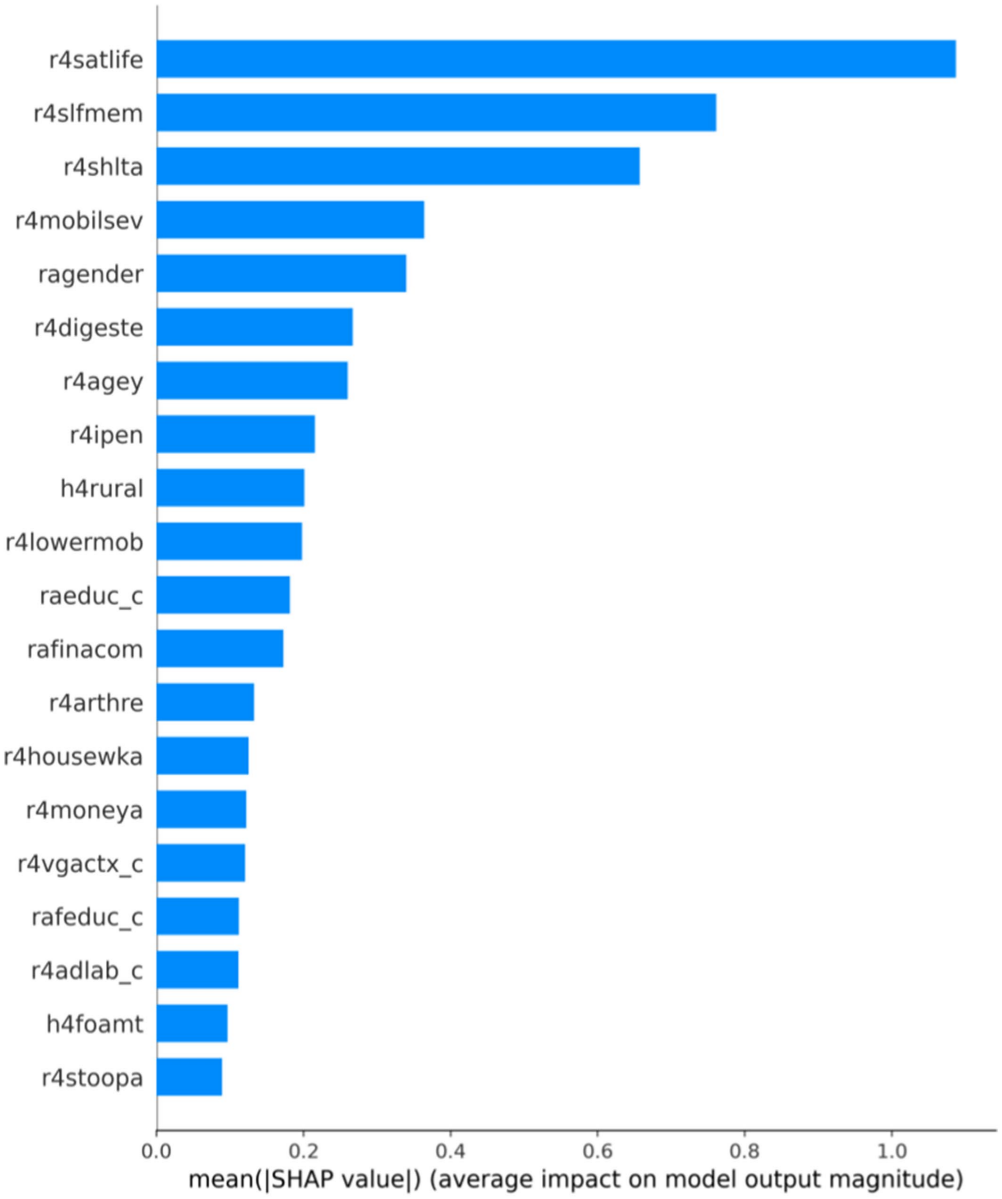
Bar plot of feature importance based on CatBoost-SHAP.

We used SHAP to find the critical influencing factors at the whole dataset level and determine the importance of each variable. As shown in Table 2, the top three significant impact characteristics are r4satlife, r4slfmem, and r4shlta. These three crucial factors were essential features of the depression prediction model, and the SHAP values were, respectively, 1.0871, 0.7614, and 0.6572. Based on the SHAP feature importance bar plot of the CatBoost model, we can more intuitively understand the variable dimensions that play an important role. As shown in Figure 2, the influence values of the top three crucial factors, such as r4satlife, r4slfmem, and r4shlta, were all above 0.6.

**Table 2:** Order of key influencing factors.

Variable	Feature importance value	Meanings
r4satlife	1.0871	Satisfied with life
r4slfmem	0.7614	Self-reported memory
r4shlta	0.6572	Self-reported health
r4mobilsev	0.3636	7 item mobility
ragender	0.3395	Gender
r4digeste	0.2665	Ever had stomach/digestive disease
r4agey	0.2600	Age in years
r4ipen	0.2152	Income: total pension income
h4rural	0.2007	Lives in rural or urban
r4lowermob	0.1974	Lower body mobility
raeduc_c	0.1809	Education
rafinacom	0.1728	Financial status compared to the average family in the same
r4arthre	0.1329	Ever had arthritis disease
r4housewka	0.1254	Some Diff-cleaning house
r4moneys	0.1221	Managing money
r4vgactx_c	0.1205	Days/wk. vigorous physical activity or exercise
rafeduc_c	0.1123	Father’s education
r4adlab_c	0.1115	6-item summary
h4foamt	0.0968	The number of transfers from others
r4stoopa	0.0891	Stooping, kneeling, or crouching

**Figure 3:**
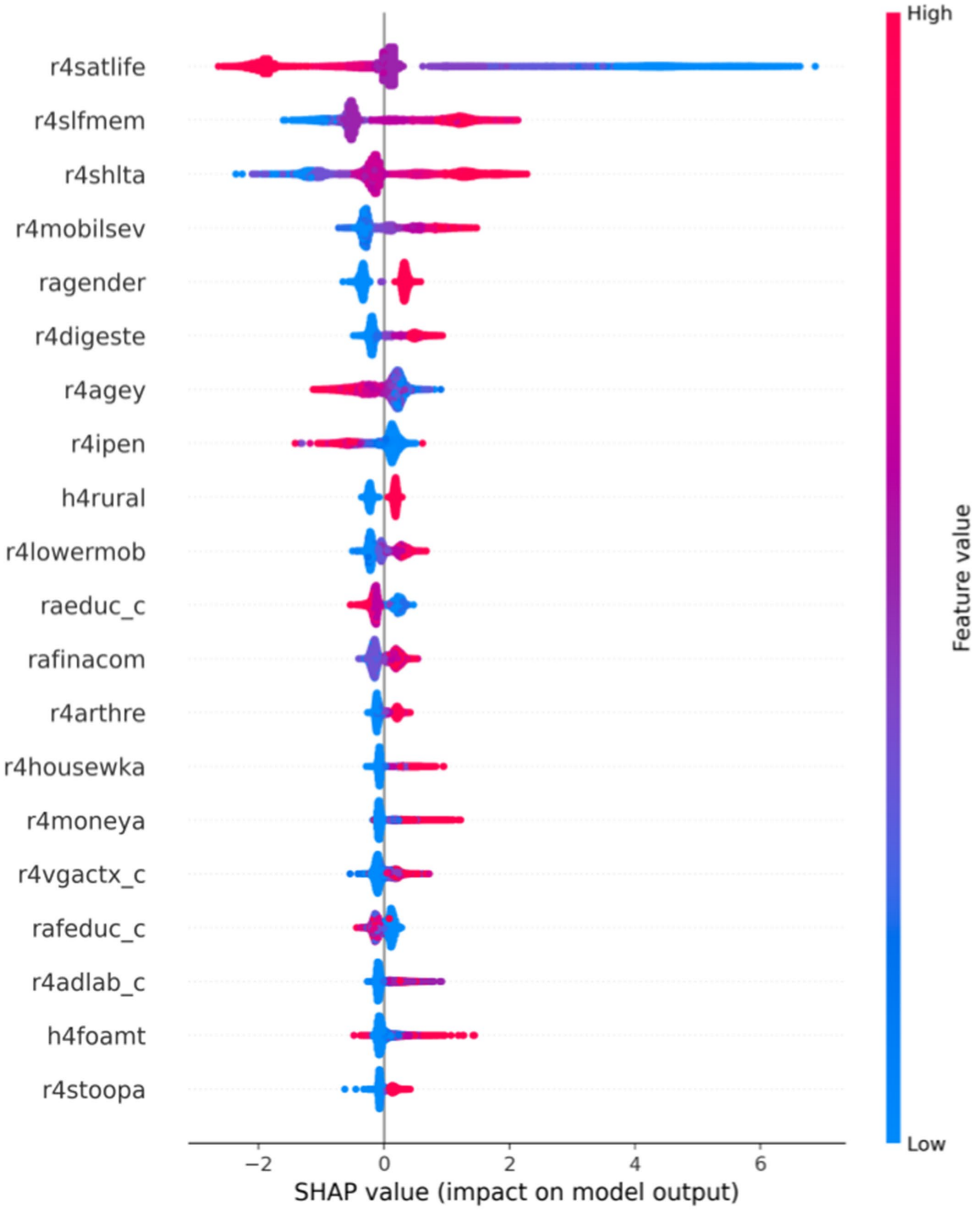
Beeswarm plot of effects of features based on CatBoost-SHAP.

SHAP beeswarm plot provides the degree of importance of the variable and the positive and negative impact on the CatBoost model’s prediction results. In Figure 3, each row represents a variable, and the abscissa is the SHAP value, showing the importance ranking of each feature SHAP value from top to bottom. Each point represents a sample. The redder the color, the larger the feature itself, and the bluer the color, the smaller the feature itself. When the SHAP value is positive, it indicates increasing the model’s prediction results; in contrast, when the SHAP value is negative, the negative impact is on the model’s output. According to the three crucial factors in Figure 3, r4satlife had the most significant effect on outcomes, indicating that greater satisfaction values decreased the probability of depression and that satisfaction and depression were negatively correlated. The scores of r4slfmem and r4shlta ranged from 1 for excellent to 5 for very poor, meaning that higher respondent scores indicate worse self-rated memory and health. The results from Figure 3 showed that higher scores of self-reported memory and self-reported health increased the risk of depression. Thus, self-reported memory and health were positively associated with depression.

**Figure 4:**
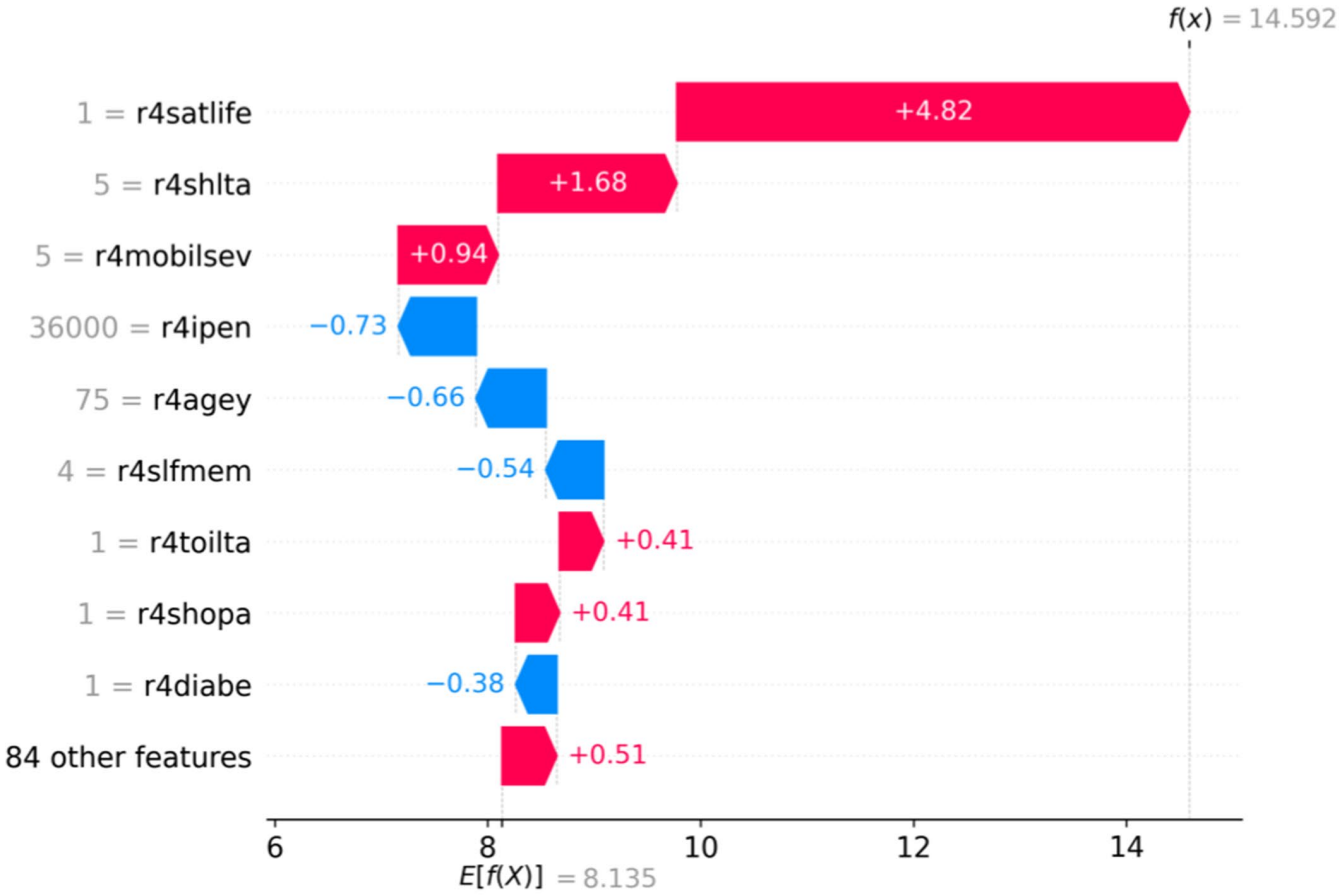
Analysis of SHAP values for single-sample full features.

**Figure 5:**
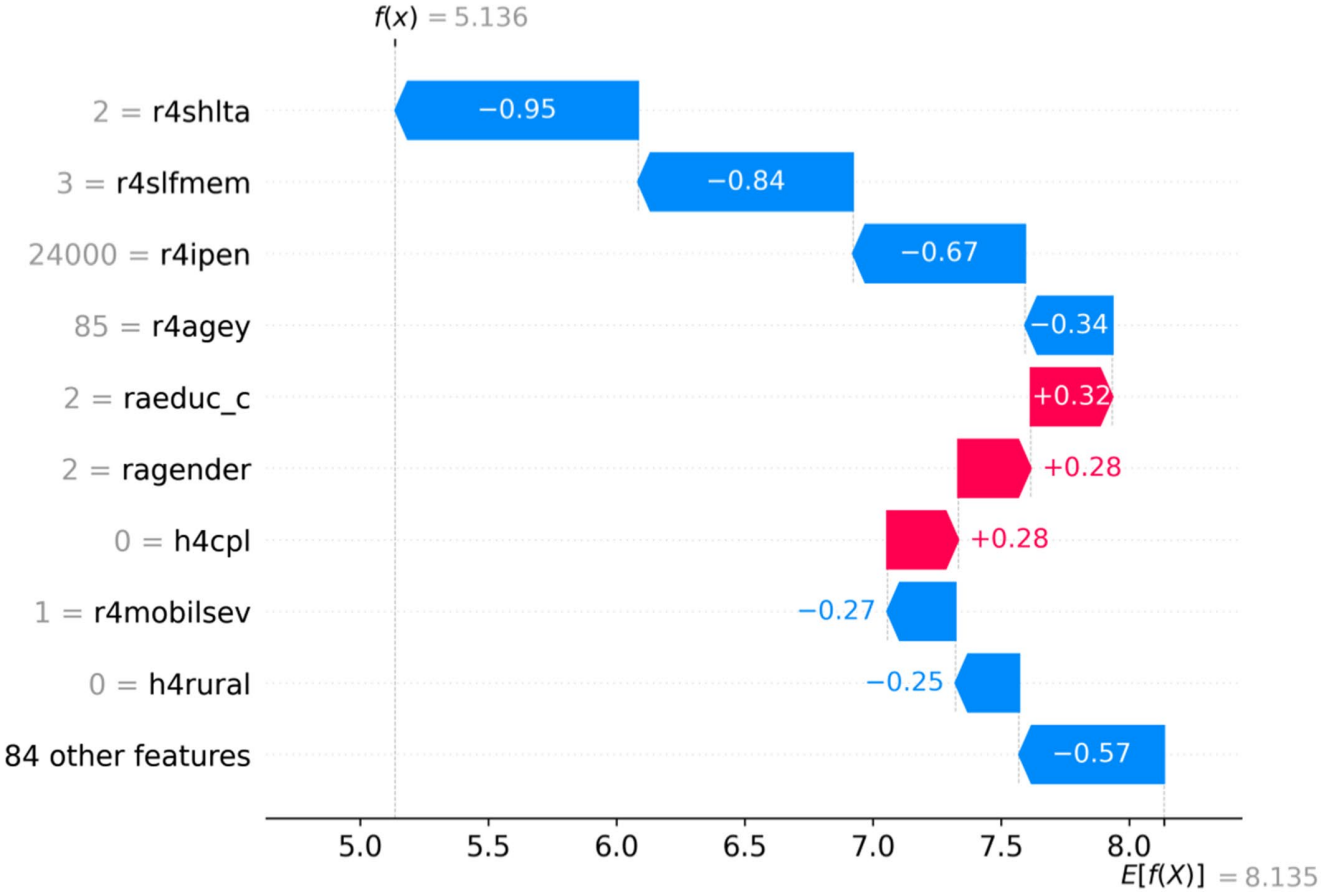
SHAP values analysis of single-sample full features.

In this study, two samples were randomly selected to perform SHAP analysis to analyze the instance-level influencing factors and discover individual heterogeneity. As shown in Figures 4, 5, the vertical axis represents different feature values; the horizontal axis represents SHAP values, and E (f(x)) represents the expectation of the predicted value of all samples. The red section indicates a positive effect on predicted depressive values, and the blue section suggests a negative impact. Figure 4 demonstrates that the expected value of a sample f(x) is 14.592, much higher than the E (f(x)), indicating that the individual’s depression is higher than the average level. According to the SHAP interpretation, the reasons why this sample was predicted as a higher level of depression were mainly influenced by variables such as r4satilfe, r4shlta, r4moblisev, r4toilta, and r4shopa. In Figure 5, the predicted value of sample f(x) is 5.136, indicating that the individual is lower than the average depression level. This lower level of depression is influenced by variables such as r4shlta, r4slfmem, r4ipen, r4agey, r4mobilsev, and r4rural. Therefore, under the SHAP individual case analysis, the crucial influencing factors of different samples are different, which reflects the individual heterogeneity well.

### 3.3 ALE analysis results

**Figure 6:**
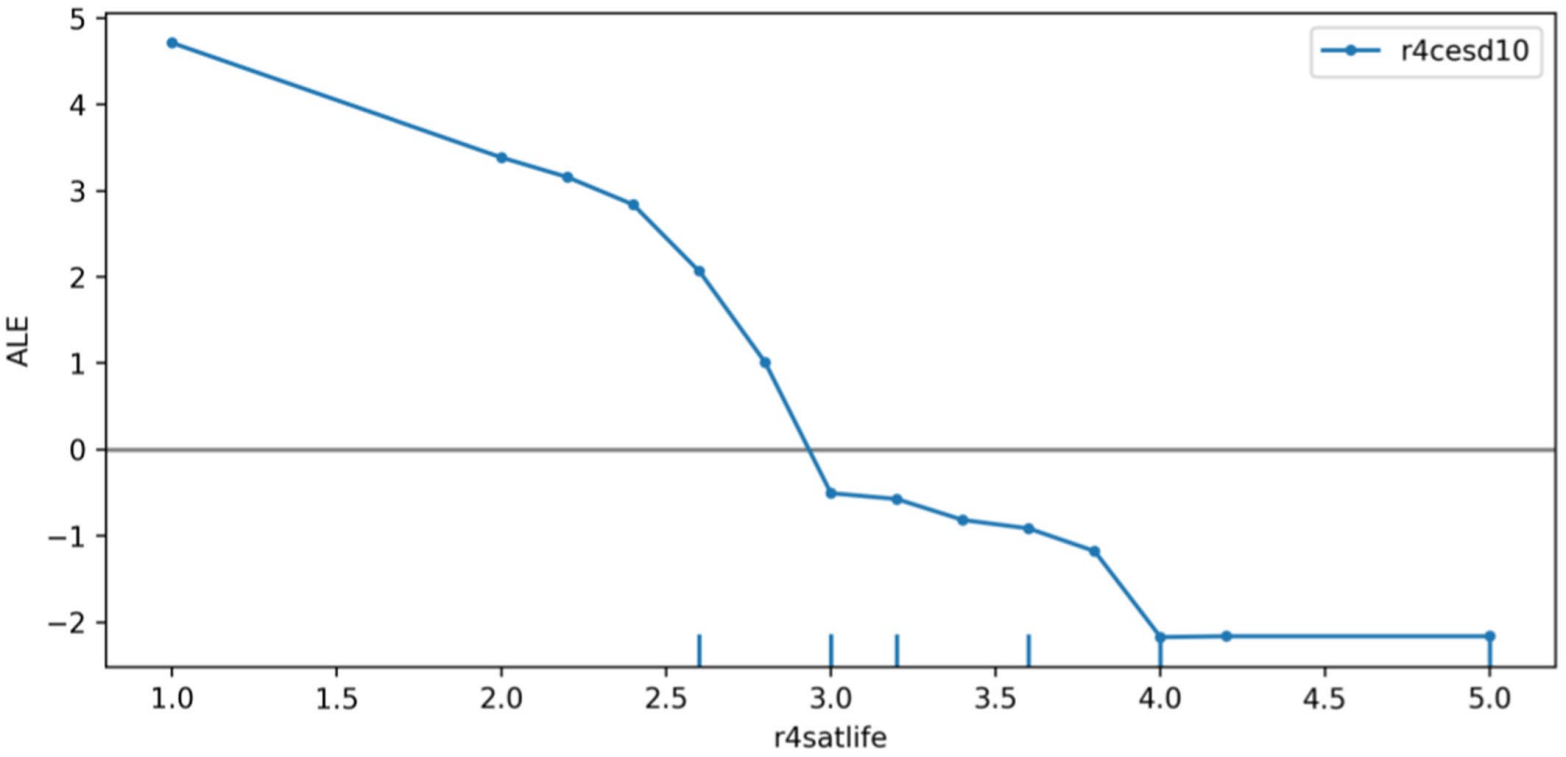
ALE plot of r4satlife effect on depression.

**Figure 7:**
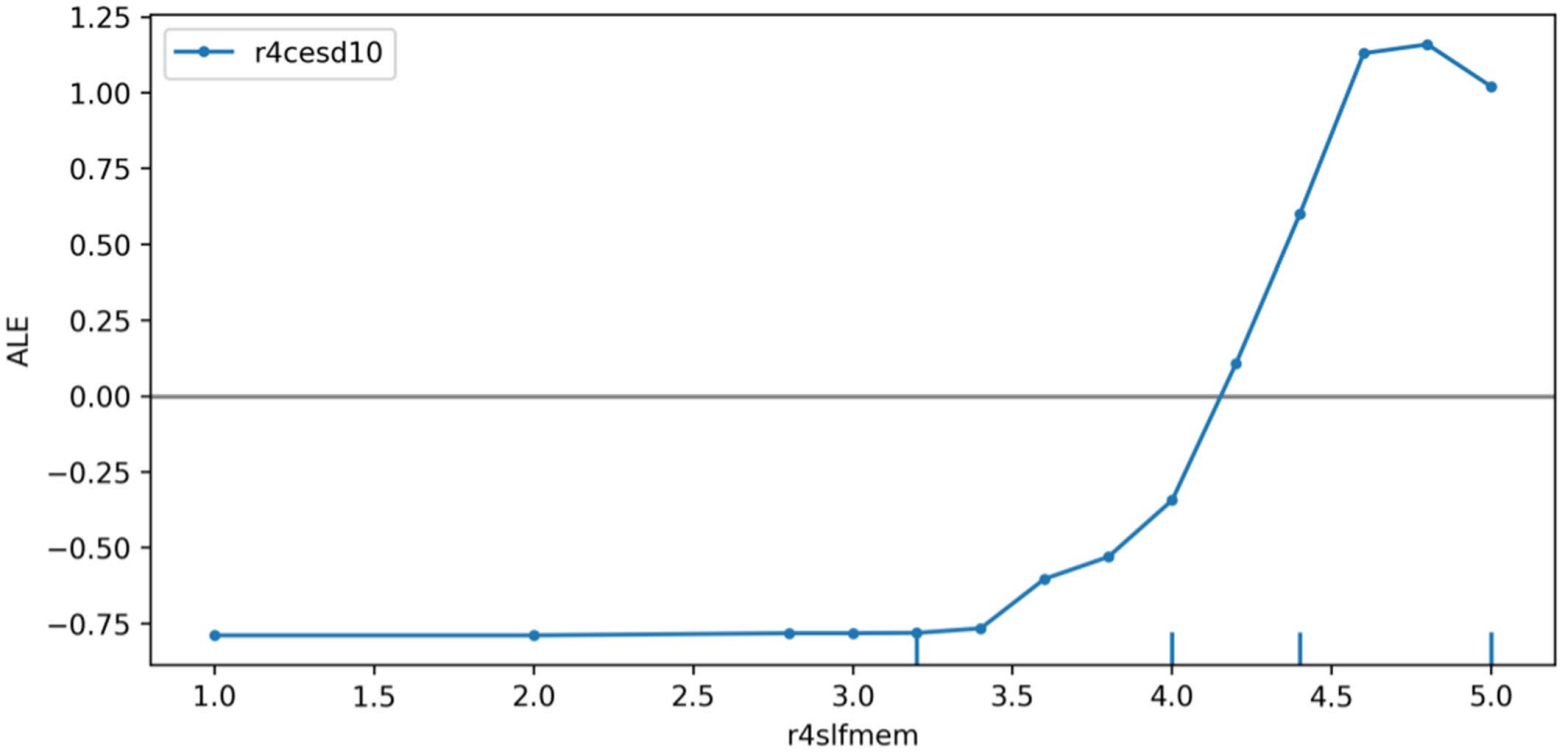
ALE plot of r4slfmem effects on depression.

**Figure 8:**
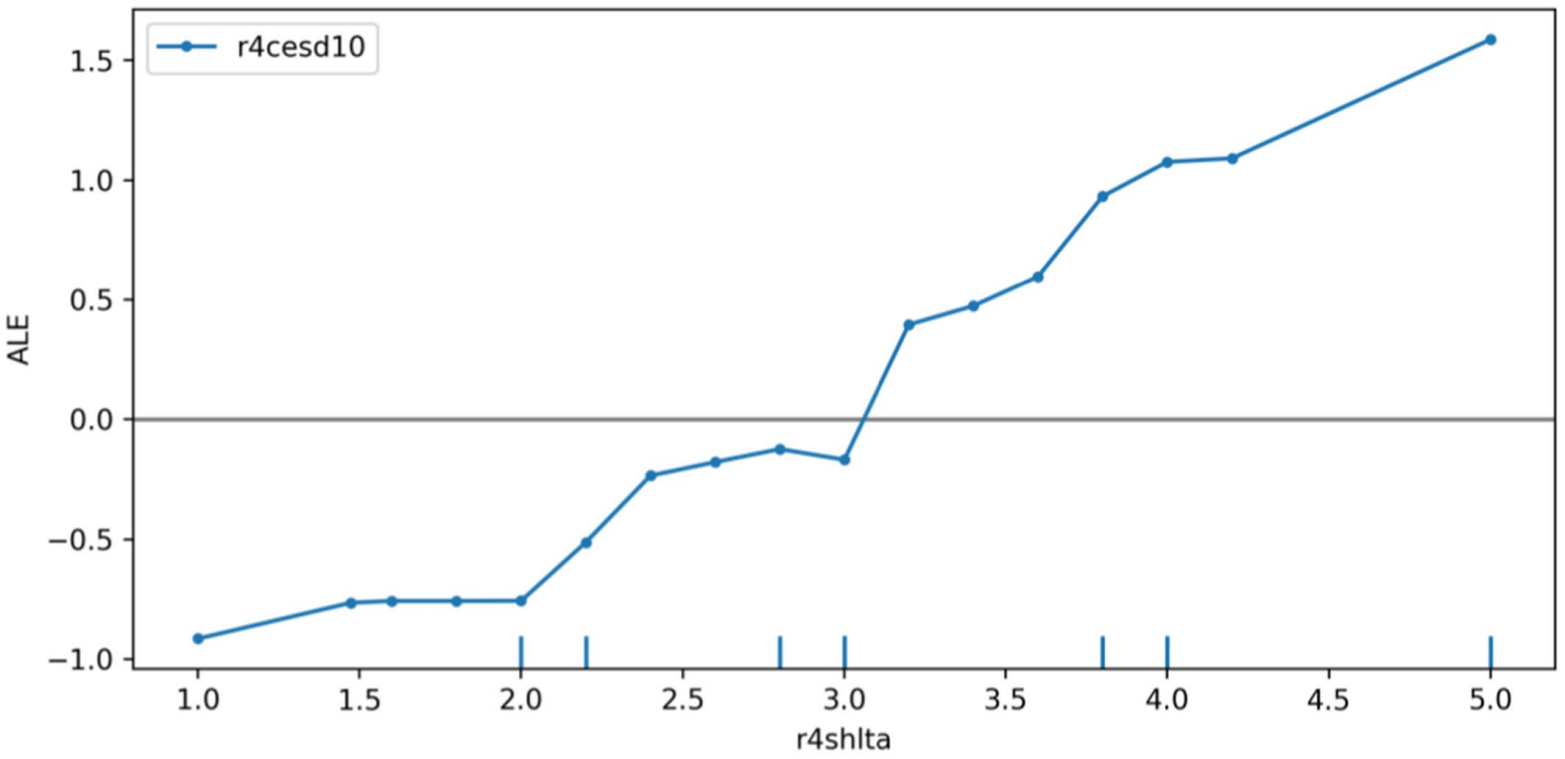
ALE plot of r4shlta effects on depression.

We plotted the ALE of the depression prediction model based on the r4satlife, r4slfmem, and r4shlta. Figure 6 shows that r4satlife has a strong negative impact on the prediction of depression, and the prediction value will decrease with the increase in life satisfaction. Since the scores on r4slfmem from 1 for excellent to 5 for very poor, Figure 7 shows that higher scores of respondents’ self-reported memory had a strong positive effect on depression prediction, and the prediction value will increase with the higher self-reported memory. That is, the worse the respondents’ self-reported memory, the easier it is to predict the risk of depression. However, when the self-reported memory was greater than 4.7, the impact of the higher memory value on the increased predictive value of depression was weakened. Since the scores on r4shlta from 1 for excellent to 5 for very poor, Figure 8 shows that higher scores of respondents’ self-reported health status of the individuals also had a strong positive impact on the prediction of depression, and the predicted value overall increased with the higher self-reported health value. That is, the worse the respondents’ self-reported health, the easier it is to predict the risk of depression.

### 3.4 Robustness test

To ensure the reliability of the findings of this study, we also conducted the robustness test. There are three main parts of the test. First, the samples with missing values of the dependent variable are excluded. In the baseline study, we used KNN to interpolate the missing values of the dependent variable to retain more samples. In the robustness test, we exclude these samples with missing values for the depression outcome variable and look at the performance of each machine learning model. The results are shown in Supplementary Table S2, where it can be seen that due to the reduced sample size, there are relatively fewer patterns of data to learn, and the error of each model has increased. However, Catboost is still the comparatively better model. The three most important variables remain r4satlife , r4shlta , and r4slfmem .

Second, due to the 93 input variables in the benchmark experiment, there is a more severe problem of multicollinearity. To deal with this concern, this study adopts the Recursive Feature Elimination (RFE) method (Guyon et al., 2002), which sequentially removes the input features with the highest Variance Inflation Factor (VIF) values until the VIFs of all the features are less than 5. As shown in Supplementary Figure S2, during this process, the performance of the CatBoost model retraining and validation are recorded. It can be found that the multicollinearity problem is no longer serious after the input features are reduced to 58. CatBoost’s performance decreases throughout the RFE process but still performs relatively best with the original 93 inputs. This indicates that the multicollinearity problem is not severe for CatBoost and is consistent with the characteristics of machine learning methods such as tree-based models (Zhao et al., 2019; Chan et al., 2022).

Third, due to the stochastic nature of machine learning, this study uses a 5-fold cross-validation (CV) approach to compare the comprehensive performance of each model in the baseline experiment. The results record the mean and standard deviation of the performance of all models in cross-validation, and it can be seen that the CatBoost model is still robust and has about 14% improvement over the LR model in the critical *R*^2^ measurement. The detailed results of the robustness test can be found in the Supplementary material.

## 4 Discussion

In this study, based on data from the fourth wave of Harmonized CHARLS, we obtained demographic variables, health status, cognitive, income, family structure, stress, life satisfaction, and CESD in a total of 25,586 middle-aged and older people sample. Five machine learning and traditional models showed that the former was the best in predicting the risk of depression, consistent with earlier related studies (Ay et al., 2019). Moreover, further comparison found that the CatBoost model more accurately predicted the risk of depression in older adults. Therefore, based on the CatBoost model, the SHAP method was used to analyze the crucial factors to obtain the global interpretation of the whole sample set and the feature importance ranking of the model. There were three significant impact characteristics: life satisfaction, self-reported memory, and self-reported health. Similarly, previous studies also found that there is considerable heterogeneity in depressive symptoms in older adults, but cognition and self-reported memory are considered to predict the essential characteristics of ‘depressive symptoms events increased trajectory’ and ‘chronic symptoms trajectory’ (Lin et al., 2023), self-reported memory presented was also a vital influence feature obtained in this study.

The SHAP method analysis found that life satisfaction was one of the most critical key impact features that predicted depression levels. Prior studies have also shown that the higher the level of depression in middle-aged and older adults, the lower the life satisfaction, which is the strongest negative predictor of depression in older adults (Yoo et al., 2016). Lee’s study of older Koreans found that life satisfaction increased significantly over time, and the level of depression decreased (Lee et al., 2020). These are consistent with the findings presented in the present study. We considered the following reasons: on the one hand, life satisfaction is an evaluation indicator of subjective well-being and is an important part of improving the quality of life of older adults (Chachamovich et al., 2007). A study showed that subjective well-being and depression were negatively correlated with each other (Bartels et al., 2013). On the other hand, life satisfaction was one of the important characteristics of individuals with chronic and highly stable depression trajectories, indicating that life satisfaction and the incidence of depression are highly related (Kaup et al., 2016).

In this study, self-reported memory is confirmed to be a more critical influencing factor. Sun’s study found that memory was significantly negatively associated with depression (Sun et al., 2019). Zhou’s study also found that participants with depressive symptoms all had poor cognitive function (Zhou et al., 2021). We considered the following reasons: On the one hand, the deterioration of mental function is related to depression. Episodic memory deteriorated in answering structured questions (but not free recollection of past events) among depressed patients (Wachowska et al., 2022).

On the other hand, cognitive impairment appears to be the core pathological symptom of depression, and cognitive impairment may appear before depression parameters (Maramis et al., 2021). Some argue that cognitive symptoms should be viewed as a separate dimension and an important target for any treatment that has already started (Planchez et al., 2019). Therefore, in this study, self-reported memory is considered to be a more important influencing factor in predicting depression.

Self-reported health is also confirmed to be a more important influencing factor. Kuchibhatla’s study found that the reduction in depressive symptoms is strongly associated with health (Kuchibhatla et al., 2012). Kaup’s study found that good education, better health, fewer stressful events, and a more extensive social network would reduce the incidence of depressive symptoms (Kaup et al., 2016). Chan’s study found that a history of self-perceived health and perceived low cost in older men are important risk factors for depression (Chan and Zeng, 2011). Wen’s study found that individuals who perceived their health status as excellent had a 62% lower risk of depression compared with those who perceived their health as poor (Wen et al., 2019). The reason is that self-rated health was associated with both objective health status and components of psychological perceptions. It has been found to be associated with depression factors that share the same psychological and biological mechanisms (Peleg and Nudelman, 2021). Therefore, in this study, self-reported health is considered to be a more important influencing factor in predicting depression.

Based on the entire interpretable machine learning framework, this study identified the most critical factors affecting depression levels in a hypothesis-free manner, excluding possible interference from confounding factors, demonstrating the most crucial position of life satisfaction. Meanwhile, we randomly selected two respondents and explained the key factors affecting individual depression levels, namely, finding individual heterogeneity. In further research, using the ALE method, we found the depression prediction model diagram of some essential factors, so we can also change the level of depression and improve the quality of life by controlling for some related variables.

This study has three innovations but also has three limitations. First, we did not use the longitudinal data, only the fourth wave of the Harmonized CHARLS data. Second, the study did not include variables such as finance, housing, health care, and insurance. Third, we did not adopt more complex parameter adjustment methods, most of which used the default configuration, which has some limitations on the applicability of the challenging depression prediction task. In future research, we need to develop more advanced integration models.

## Data availability statement

This analysis uses data or information from the Harmonized CHARLS dataset and Codebook, Version D as of June 2021 developed by the Gateway to Global Aging Data. The development of the Harmonized CHARLS was funded by the National Institute on Aging (R01 AG030153, RC2 AG036619, R03 AG043052). For more information, please refer to https://g2aging.org/. The CHARLS data can be accessed at: https://charls.charlsdata.com/pages/Data/harmonized_charls/en.html. For more details and code for this study, please contact the corresponding author.

## Author contributions

RL: Conceptualized, Methodology, Writing – original draft. XW: Conceptualized, Methodology, Writing – review & editing. LL: Conceptualized, Methodology, Writing – review & editing. YY: Conceptualized, Writing – review & editing.
