# Supplementary material for: Identifying the most crucial factors associated with depression based on interpretable machine learning: a case study from CHARLS
Source: Front Psychol. 2024 Jul 25;15:1392240. doi: 10.3389/fpsyg.2024.1392240 (PMC11306142; doi:10.3389/fpsyg.2024.1392240)
Supplement: Supplementary file 1 [file Table_1.docx]

Supplementary Material

This Supplementary Material focuses on presenting the results of the variables' descriptive statistics in Section 2.2 with the robustness tests in Section 3.4 of the main text. The basic descriptive statistics for the 93 input variables and one output outcome variable used in the baseline experiment of this study are shown in Table S1 (N=25586).

Table S1. Baseline characteristics of the CHARLS participants

| **Variables** | **Distribution** |
| --- | --- |
| **Demographic** | |
| **Rabplace_C**: Birth place, *N* (%) |  |
| 1. interview | 11710 (45.8) |
| 1. Another village but same province as the interview place | 10752 (42) |
| 1. Another village in another province | 2723 (10.6) |
| 1. Abroad | 7 (.0) |
| **Raeduc_C**: Education level, *N* (%) |  |
| 1. No formal education, illiterate | 6256 (24.5) |
| 1. Did not finish primary school but capable of reading | 3917 (15.3) |
| 1. Sishu | 104 (4) |
| 1. Elementary school | 7200 (28.1) |
| 1. Middle school | 4828 (18.9) |
| 1. High school | 1978 (7.7) |
| 1. Vocational school | 606 (2.4) |
| 1. Two/Three Year College/Associate degree | 398 (1.6) |
| 1. Four Year College degree | 237 (0.9) |
| 1. Master/PhD | 18 (0.1) |
| **R4mnev**: Never Married, *N* (%) |  |
| 1. No | 19698 (77) |
| 1. Yes | 118 (0.5) |
| **Ragender**: Gender, *N* (%) |  |
| 1. Male | 12334 (48.2) |
| 1. Female | 13244 (51.8) |
| **R4hukou,** *N* (%) |  |
| 1. Agricultural hukou | 13927 (54.4) |
| 1. Non-agricultural hukou | 3680 (14.4) |
| 1. Unified residence hukou | 491 (1.9) |
| 1. Do not have hukou | 25 (0.1) |
| **H4cpl**: A couple household, *N* (%) |  |
| 1. Not coupled | 3499 (13.7) |
| 1. Coupled | 16317 (63.8) |
| **H4rural**:Urban or Rural, *N* (%) |  |
| 1. Rural | 14242 (55.7) |
| 1. Urban | 11344 (44.3) |
| **R4agey**: Age, mean (SD) | 61.44 (10.41) |
| **Health status variable** | |
| **R4psyche**: Whether the respondent reported having any emotional, nervous, or psychiatric problems, *N* (%) |  |
| 1. No | 19150 (74.8) |
| 1. Yes | 647 (2.5) |
| **R4asthmae**: Whether the respondent reported having asthma, *N* (%) |  |
| 1. No | 18629 (72.8) |
| 1. Yes | 1168 (4.6) |
| **R4memrye**: Whether the respondent reported having memory-related disease, *N* (%) |  |
| 1. No | 19002 (74.3) |
| 1. Yes | 794 (3.1) |
| **R4cancre**: Whether the respondent reported having cancer, *N* (%) |  |
| 1. No | 19345 (75.6) |
| 1. Yes | 449 (1.8) |
| **R4kidneye**: Whether the respondent reported having kidney disease, *N* (%) |  |
| 1. No | 17829 (69.7) |
| 1. Yes | 1965 (7.7) |
| **R4stroke**: Whether the respondent reported having stroke, *N* (%) |  |
| 1. No | 18368 (71.8) |
| 1. Yes | 1425 (5.8) |
| **R4livere**: Whether the respondent reported having liver disease, *N* (%) |  |
| 1. No | 18418 (72) |
| 1. Yes | 1374 (5.4) |
| **R4diabe**: Whether the respondent reported having diabetes disease, *N* (%) |  |
| 1. No | 17254 (67.4) |
| 1. Yes | 2538 (9.9) |
| **R4lung**: Whether the respondent reported having lunge disease, *N* (%) |  |
| 1. No | 16736 (65.4) |
| 1. Yes | 3055 (11.9) |
| **R4digeste**: Whether the respondent reported having digestive disease, *N* (%) |  |
| 1. No | 13834 (54.1) |
| 1. Yes | 5951 (23.3) |
| **R4hearte**: Whether the respondent reported having heart disease, *N* (%) |  |
| 1. No | 15884 (62.1) |
| 1. Yes | 3899 (15.2) |
| **R4hibpe**: Whether the respondent reported having high blood pressure disease, *N* **(%)** |  |
| 1. No | 12217 (47.7) |
| 1. Yes | 7563 (29.6) |
| **R4dyslipe**: Whether the respondent reported having dyslipidemia disease, *N* (%) |  |
| 1. No | 15438 (60.3) |
| 1. Yes | 4341 (17) |
| **R4arthre**: Whether the respondent reported having arthritis disease, *N* (%) |  |
| 1. No | 12092 (47.3) |
| 1. Yes | 7687 (30) |
| **R4drinkev**: Ever drinks any alcohol, *N* (%) |  |
| 1. No | 10400 (40.6) |
| 1. Yes | 9342 (36.5) |
| **R4smokev**: Ever smoking, *N* (%) |  |
| 1. No | 11439 (44.7) |
| 1. Yes | 8295 (32.4) |
| **R4vgact_c**: Any vigorous physical activity, *N* (%) |  |
| 1. No | 13511 (52.8) |
| 1. Yes | 6221 (24.3) |
| **R4vgactx_c**: The number of days of vigorous activity, *N* (%) |  |
| 0 | 13511 (52.8) |
| 1 | 366 (1.4) |
| 2 | 645 (2.5) |
| 3 | 641 (2.5) |
| 4 | 391 (1.5) |
| 5 | 452 (1.8) |
| 6 | 129 (0.5) |
| 7 | 3597 (14.1) |
| **R4mdact_c**: Any moderately physical activity, *N* (%) |  |
| 0.No | 10107 (39.5) |
| 1. Yes | 9625 (37.6) |
| **R4mdactx_**c: The number of days of moderate activity, *N* (%) |  |
| 0 | 10107 (39.5) |
| 1 | 444 (1.7) |
| 2 | 838 (3.3) |
| 3 | 1040 (4.1) |
| 4 | 452 (1.8) |
| 5 | 429 (1.7) |
| 6 | 129 (0.5) |
| 7 | 6293 (24.6) |
| **R4ltact_c**: Any light physical activity, *N* (%) |  |
| 1. No | 3447 (13.5) |
| 1. Yes | 16285 (63.6) |
| **R4ltactx_c**: The number of days of light activity, *N* (%) |  |
| 0 | 3447 (13.5) |
| 1 | 208 (0.8) |
| 2 | 492 (1.9) |
| 3 | 673 (2.6) |
| 4 | 385 (1.5) |
| 5 | 492 (1.9) |
| 6 | 133 (0.5) |
| 7 | 13902 (54.3) |
| **R4mealsa**: Preparing meals, *N* (%) |  |
| 1. Not reported any problems | 17467 (68.3) |
| 1. Reported some difficulty | 2254 (8.8) |
| **R4phonea**: Making phone calls, *N* (%) |  |
| 1. Not reported any problems | 16480 (64.4) |
| 1. Reported some difficulty | 2094 (8.2) |
| **R4moneya**: Managing money, *N* (%) |  |
| 1. Not reported any problems | 17417 (68.1) |
| 1. Reported some difficulty | 2304 (9) |
| **R4medsa**: Taking medications, *N* (%) |  |
| 1. Not reported any problems | 18567 (72.6) |
| 1. Reported some difficulty | 1154 (4.5) |
| **R4shopa**: Shopping, *N* (%) |  |
| 1. Not reported any problems | 17825 (69.7) |
| 1. Reported some difficulty | 1896 (7.4) |
| **R4housewka**: Cleaning house, *N* (%) |  |
| 1. Not reported any problems | 16814 (65.7) |
| 1. Reported some difficulty | 2907 (11.4） |
| **R4lowermob**: Lower body mobility, *N* (%) |  |
| 0 | 8117 (31.7) |
| 1 | 4334 (16.9) |
| 2 | 2934 (11.5) |
| 3 | 2865 (11.2) |
| 4 | 1206 (4.7) |
| **R4walk1kma**: Walking 1KM, *N* (%) |  |
| 1. Not reported any problems | 15733 (61.5) |
| 1. Reported some difficulty | 3723 (14.6) |
| **R4chaira**: Getting up from a chair, *N* (%) |  |
| 1. Not reported any problems | 13532 (52.9) |
| 1. Reported some difficulty | 5924 (23.2) |
| **R4mobilsev**: 7 item mobility, *N* (%) |  |
| 0 | 7636 (29.8) |
| 1 | 3975 (15.5) |
| 2 | 2610 (8.4) |
| 3 | 2161 (10.2) |
| 4 | 1351 (5.3) |
| 5 | 906 (3.5) |
| 6 | 536 (2.1) |
| 7 | 281 (1.1) |
| **R4dressa**: Dressing, *N* (%) |  |
| 0. Not reported any problems | 18076 (70.6) |
| 1. Reported some difficulty | 1379 (5.4) |
| **R4uppermob**: 3-item summary of any difficulty with upper-body mobility activities, *N* (%) |  |
| 0 | 14521 (56.8) |
| 1 | 3180 (12.4) |
| 2 | 1268 (5) |
| 3 | 486 (1.9) |
| **R4stoopa:** Stooping, kneeling, or crouching, *N* (%) |  |
| 0. Not reported any problems | 12372 (48.4) |
| 1. Reported some difficulty | 7083 (27.7) |
| **R4adlfive**: 5-item summary of any difficulty with activities of daily living, *N* (%) |  |
| 0 | 16034 (62.7) |
| 1 | 1626 (6.4) |
| 2 | 727 (2.8) |
| 3 | 405 (1.6) |
| 4 | 354 (1.4) |
| 5 | 309 (1.2) |
| **R4adlab_c**: 6-item summary, *N* (%) |  |
| 0 | 15818 (61.8) |
| 1 | 1693 (6.6) |
| 2 | 761 (3) |
| 3 | 426 (1.7) |
| 4 | 311 (1.2) |
| 5 | 249 (1) |
| 6 | 197 (0.8) |
| **R4adla_c**: 4-item summary, *N* (%) |  |
| 0 | 16859 (65.9) |
| 1 | 1267 (5) |
| 2 | 573 (2.2) |
| 3 | 430 (1.7) |
| 4 | 326 (1.3) |
| $\mathbf{R4armsa}$: Reaching arms above shoulder level, *N* (%) |  |
| 0. Not reported any problems | 16796 (65.6) |
| 1. Reported some difficulty | 2659 (10.4) |
| $\mathbf{R4dlmeas}$: Picking up a coin from the table, *N* (%) |  |
| 0. Not reported any problems | 18408 (71.9) |
| 1. Reported some difficulty | 1047 (4.1) |
| $\mathbf{R4lifta}$: Lifting or carrying weights over ten jins, *N* (%) |  |
| 0. Not reported any problems | 15987 (62.5) |
| 1. Reported some difficulty | 3468 (13.6) |
| $\mathbf{R4climsa}$: Climbing several flights of stairs without resting, *N* (%) |  |
| 0. Not reported any problems | 10502 (41) |
| 1. Reported some difficulty | 8953 (35) |
| $\mathbf{R4toilta}$: Using the toilet, *N* (%) |  |
| 0. Not reported any problems | 17205 (67.2) |
| 1. Reported some difficulty | 2249 (8.8) |
| **R4eata**: Eating, *N* (%) |  |
| 0. Not reported any problems | 18912 (73.9) |
| 1. Reported some difficulty | 542 (2.1) |
| **R4urina**: Controlling urination and defecation, *N* (%) |  |
| 0. Not reported any problems | 18546 (72.5) |
| 1. Reported some difficulty | 908 (3.5) |
| **R4batha**: Bathing and showering, *N* (%) |  |
| 0. Not reported any problems | 17779 (69.5) |
| 1. Reported some difficulty | 1675 (6.5) |
| **R4beda**: Getting in and out of bed, *N* (%) |  |
| 0. Not reported any problems | 18043 (70.5) |
| 1. Reported some difficulty | 1411 (5.5) |
| **R4shlta**: Self-report of health, *N* (%) |  |
| 1. Very good | 2229 (8.7) |
| 1. Good | 2348 (9.2) |
| 1. Fair | 8938 (34.9) |
| 1. Poor | 3687 (14.4) |
| 1. Very poor | 1076 (4.2) |
|  |  |
| **Family Structure** | |
| **H4coresd**: Any children with them, *N* (%) |  |
| 1. No | 11311 (44.2) |
| 1. Yes | 8301 (32.4) |
| **Rameduc_c**: Mother'education level, *N* (%) |  |
| 1. No formal education, illiterate | 20353 (79.5) |
| 1. Did not finish primary school but capable of reading or writing | 1527 (6) |
| 1. Sishu | 285 (1.1) |
| 1. Elementary school | 1543 (6) |
| 1. Middle school | 488 (1.9) |
| 6. High school | 169 (0.7) |
| 1. Vocational school | 98 (0.4) |
| 1. Two/Three Year College/Associate degree | 13 (0.1) |
| 1. Four Year College degree | 30 (0.1) |
| 1. PhD | 2 (0) |
| **Rafeduc_c**: Education level, *N* (%) |  |
| 1. No formal education, illiterate | 13209 (51.6) |
| 1. Did not finish primary school but capable of reading or writing | 3151(12.3) |
| 1. Sishu | 2146 (8.4) |
| 1. Elementary school | 3216 (12.6) |
| 1. Middle school | 1382 (5.4) |
| 1. High school | 507 (2) |
| 1. Vocational school | 311 (1.2) |
| 1. Two/Three Year College/Associate degree | 98 (4) |
| 1. Four Year College degree | 197 (8) |
| 1. Master | 3(0) |
| 1. PhD | 1(0) |
| **H4dchild**: Total number of deceased children, *N* (%) |  |
| 0 | 17842 (69.7) |
| 1 | 1596 (6.2) |
| 2 | 266 (1) |
| 3 | 71 (0.3) |
| 4 | 30 (0.1) |
| 5 | 8 (0) |
| 6 | 1 (0) |
| 7 | 2 (0) |
| **H4child**: Number of living children, *N* (%) |  |
| 0 | 204 (0.8) |
| 1 | 3593 (14) |
| 2 | 7732 (30.2) |
| 3 | 4277 (16.7) |
| 4 | 2265 (8.9) |
| 5 | 1076 (4.2) |
| 6 | 393 (1.5) |
| 7 | 177 (0.7) |
| 8 | 66 (0.3) |
| 9 | 23 (0.1) |
| 10 | 10 (0) |
| **R4dadliv**: Father is alive, *N* (%) |  |
| 0. Deceased | 16667 (65.1) |
| 1. Alive | 2764 (10.8) |
| **H4kcnt**: Contact with their children, *N* (%) |  |
| 1. No | 1936 (7.6) |
| 1. Yes | 17468 (68.3) |
| **H4lvnear**: Live near children, *N* (%) |  |
| 0. No | 8807 (34.4) |
| 1. Yes | 10578 (41.3) |
| **R4livpar**: Number of living parents, *N* (%) |  |
| 0 | 13574 (53.1) |
| 1 | 4083 (16) |
| 2 | 1726 (6.7) |
| **Radadoccup_c**: Father's occupation , *N* (%) |  |
| 1. Agricultural | 15103 (59) |
| 1. Non-agricultural | 3811 (14.9) |
| **R4momliv**: Mother is alive, *N* (%) |  |
| 1. Deceased | 14683 (57.4) |
| 1. Alive | 4783 (18.7) |
| **H4fcamt**: Amount of transfers from children/grandchildren, mean (SD) | 4683.42 (11278.18) |
| **H4tcamt**: Amount of transfers to children/grandchildren, mean (SD) | 5249.55 (33357.27) |
| **H4fpamt**: Amount of transfers from parents/parents-in-law, mean (SD) | 226.45 (3164.22) |
| **H4tpamt**: Amount of transfers to parents/parents-in-law, mean (SD) | 970.67 (3517.25) |
| **H4foamt**: The number of transfers from others, mean (SD) | 1793.98 (8523.42) |
| **H4toamt**: The number of transfers to others, mean (SD) | 2518.18 (8302.59) |
| **H4frec**: The total amount of transfers received, mean (SD) | 6743.68 (14849.32) |
| **H4tgiv**: The total amount of transfers given, mean (SD) | 8923.82 (35304.40) |
| **H4ftot**: Net value of financial transfers, mean (SD) | -2170.63 (36953.12) |
| **R4decsib**: Number of deceased siblings, mean (SD**)** | 0.48 (0.90) |
| **R4livsib**: Number of living siblings, mean (SD) | 3.52 (1.83) |
| **R4socwk**: Social activities, *N* (%) |  |
| 1. No | 10908 (42.6) |
| 1. Yes | 8822 (34.5) |
| **Income** | |
| **R4ipen**: Private pension, mean (SD) | 5692.49 (23545.65) |
| **Stress** | |
| **Ramomdrug**: Female guardian had an alcohol and/or drug issue, *N* (%) |  |
| 0. No | 19029 (74.4) |
| 1. Yes | 142 (0.6) |
| **Rapadrug**: The respondent's guardians had alcohol and/or drug issue, *N* (%) |  |
| 1. No | 18166 (71) |
| 1. Yes | 1445 (5.6) |
| **Ramwarm_c**: Female guardian warmth summary mean score, mean (SD) | 1.790 (0.69) |
| **Ramomgrela**: Good relationship with female guardian, *N* (%) |  |
| 1. Excellent | 6474 (25.3) |
| 1. Very good | 6070 (23.7) |
| 1. Good | 3310 (12.9) |
| 1. Fair | 3107 (12.1) |
| 1. Poor | 174 (0.7) |
| **Ramomeft**: Female guardian put effort into watching over, *N* (%) |  |
| 1. A lot | 10622 (41.5) |
| 1. Some | 4024 (15.7) |
| 1. A little | 3099 (12.1) |
| 1. Not at all | 1238 (4.8) |
| **Ramomatt_c**: Received female guardian's love, *N* (%) |  |
| 1. Often | 11521 (45) |
| 1. Sometimes | 3391 (13.3) |
| 1. Rarely | 2262 (8.8) |
| 1. Never | 1762 (6.9) |
| **Radaddrug**: Male guardian had an alcohol and /or drug issue, *N* (%) |  |
| 0. No | 17293 (67.6) |
| 1. Yes | 1386 (5.4) |
| **Radadgrela**: Good relationship with male guardian, *N* (%) |  |
| 1. Excellent | 5546 (21.7) |
| 1. Very good | 5952 (23.3) |
| 1. Good | 3379 (13.2) |
| 1. Fair | 3479 (13.6) |
| 1. Poor | 232 (9) |
| **Rafinacom**: Self-rated family financial situation before age 17, *N* (%) |  |
| 1. A lot better off than them | 241 (0.9) |
| 2. Some what better off than them | 1710 (6.7) |
| 3. Same as them | 10193 (39.8) |
| 4. Somewhat worse off than them | 3189 (12.5) |
| 5. A lot worse off than them | 4676 (18.3) |
| **Rahltcom**: Health condition compared to other children, *N* (%) |  |
| 1. Much healthier | 3329 (13) |
| 1. Somewhat healthier | 3714 (14.5) |
| 1. About average | 10333 (40.4) |
| 1. Somewhat less healthy | 1599 (6.2) |
| 1. much less healthy | 1022 (4) |
| **R4chdeathe**: Experienced death of own child, *N* (%) |  |
| 1. No | 17079 (66.8) |
| 1. Yes | 2545 (9.9) |
|  |  |
| **Cognitive** | |
| **R4slfmem**: Self-reported memory, *N* (%) |  |
| 1. Excellent | 126 (0.5) |
| 1. Very good | 893 (3.5) |
| 1. Good | 1419 (5.5) |
| 1. Fair | 9893 (38.7) |
| 5. Poor | 5826 (22.8) |
| **Life Satisfaction** | |
| **R4satlife**: Life Satisfaction, *N* (%) |  |
| 1. Not at all satisfied | 577 (2.3) |
| 1. Not very satisfied | 1542 (6) |
| 1. Somewhat satisfied | 9801 (38.3) |
| 1. Very satisfied | 5303 (20.7) |
| 1. Completely satisfied | 929 (3.6) |
| **CESD-10** | |
| **R4cesd10**, mean (SD) | 8.43 (6.45) |

Table S2 shows the results of each model's re-supervised learning and prediction evaluation after excluding samples with missing values for the outcome variable $R4cesd10$ (N=18005). We also perform Optuna-based hyperparameter optimization for CatBoost at this sample size. Due to the reduction of valid information from thousands of samples, it can be seen that the effectiveness of all models decreases compared to Table 1 in the main text, but CatBoost's performances are still optimal. As shown in Figure S1, we identified critical variables based on the combination of CatBoost and SHAP again, the three most critical variables remain consistent with Figures 2 and 3 in the main text. Although the rankings of $r4shlta$ and $r4slfmem$ are swapped, it still illustrates the robustness of the top 3 crucial variables found in this study.

Table S2. Robustness test of models performance without KNN missing value filling for the outcome variable

| Model | MAE | MSE | MedAE | R2 |  |
| --- | --- | --- | --- | --- | --- |
| XGBoost | 4.1124 | 27.4458 | 3.379 | 0.314 |  |
| GBDT | 3.9761 | 25.2098 | 3.287 | 0.3699 |  |
| RF | 4.0482 | 26.0048 | 3.39 | 0.35 | |
| LightGBM | 3.9801 | 25.3631 | 3.3105 | 0.3661 | |
| CatBoost | **3.9622** | **25.135** | **3.2821** | **0.3718** |  |
| LR | 4.0574 | 25.9562 | 3.4517 | 0.3513 |  |

| 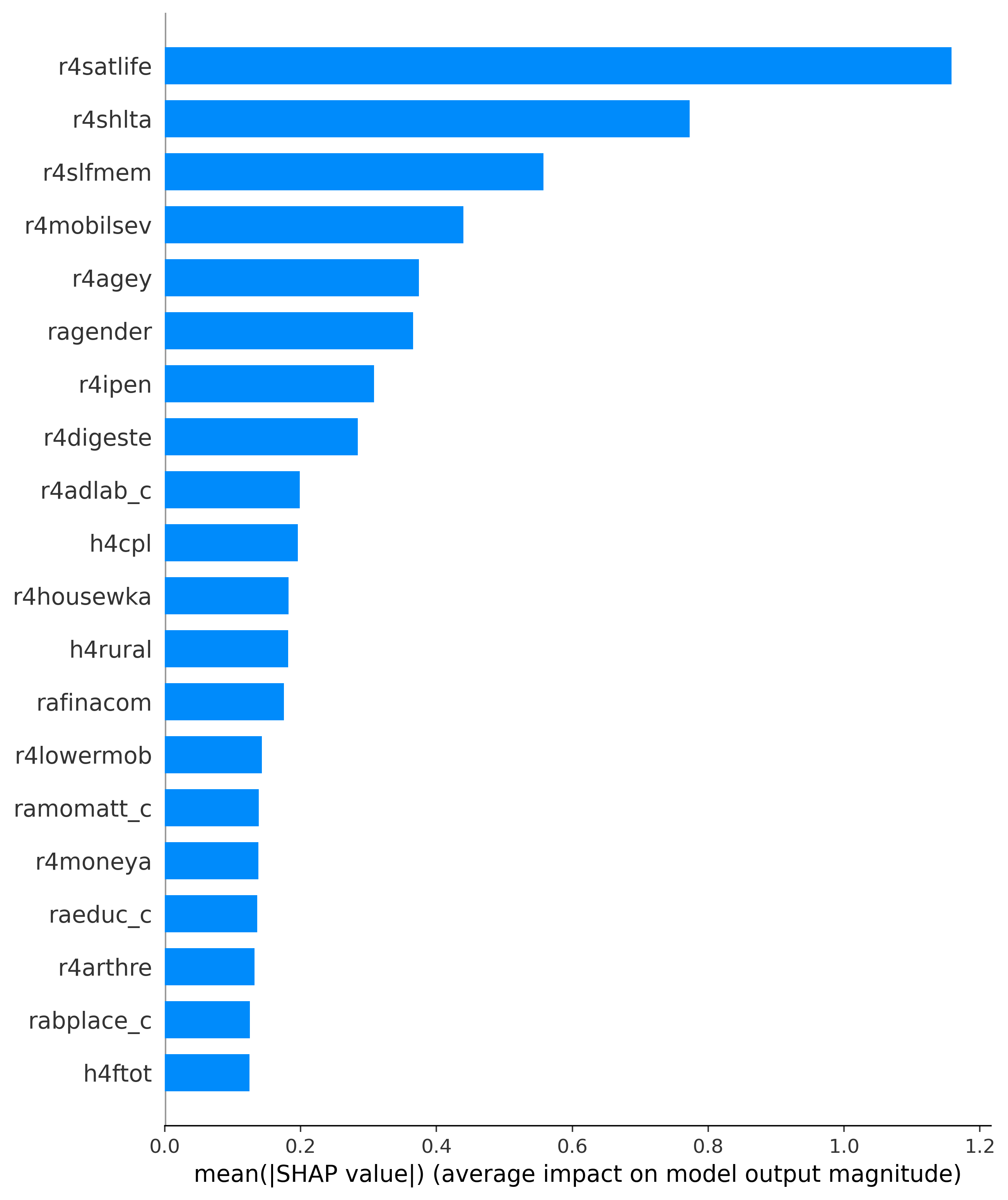   1. Bar plot | 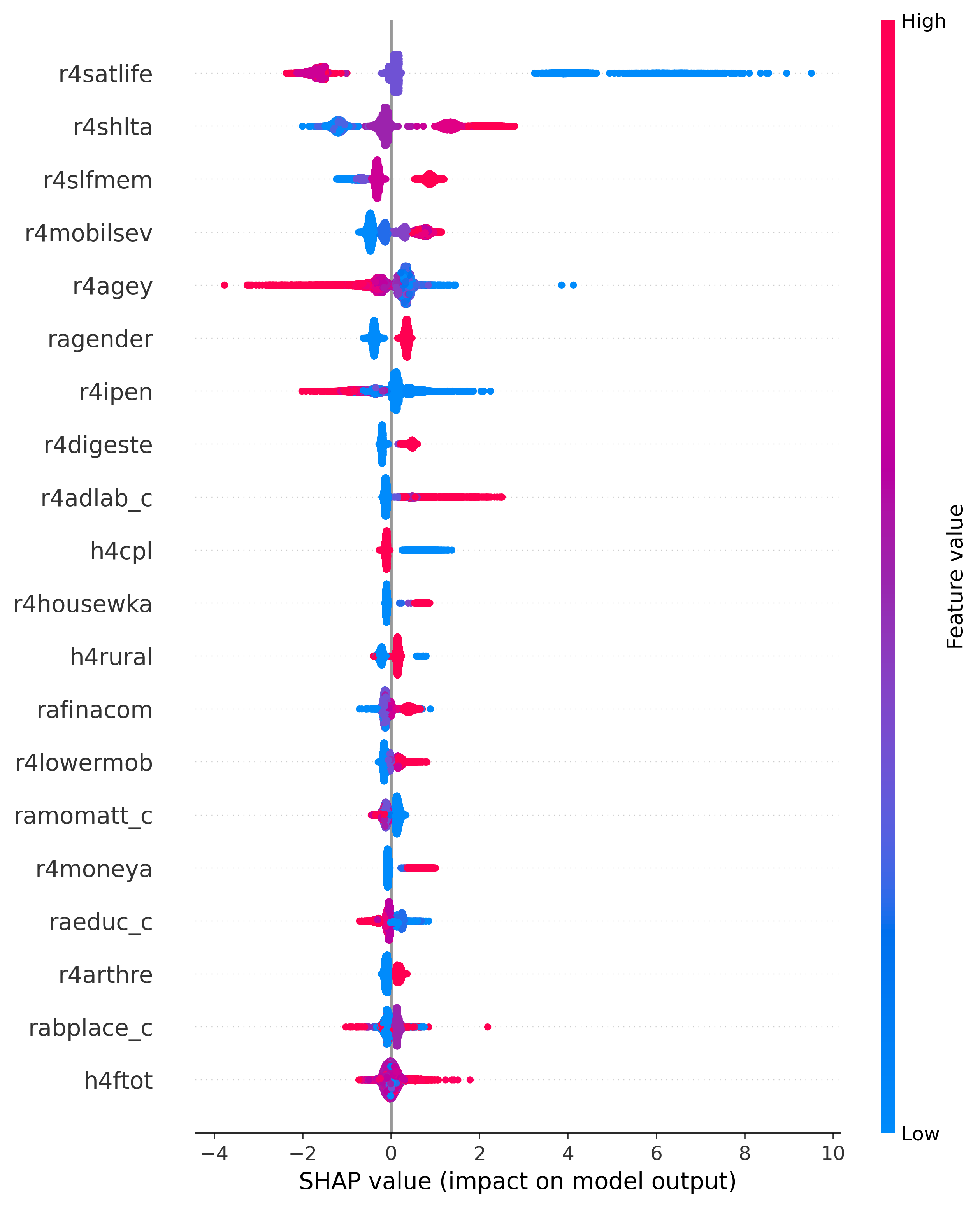   1. Beeswarm plot |
| --- | --- |

Figure S1. Critical variables identification based on CatBoost and SHAP without KNN missing value filling for the outcome variable

Figure S2 illustrates the Variance Inflation Factor (VIF) based Recursive Feature Elimination (RFE) process for the multicollinearity problem among the input variables. It can be seen that the original 93 features are gradually reduced to 58 under the criterion of VIF$<5$. The trend of multicollinearity mitigation during the RFE process and the prediction performance of CatBoost are shown in detail in Figure S3. It can be found that the more features are reduced, the worse CatBoost's performance is, which also illustrates the trade-off between the number of input features and multicollinearity.


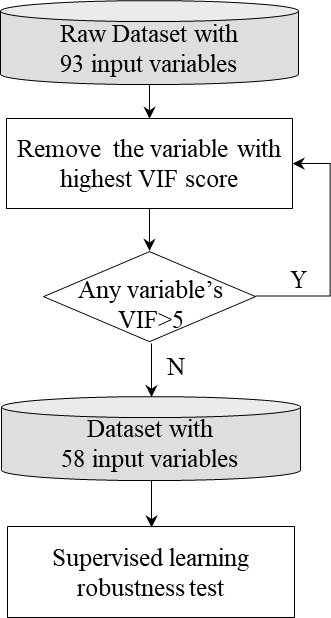


Figure S2. VIF-based RFE to deal with multicollinearity in robustness test

| 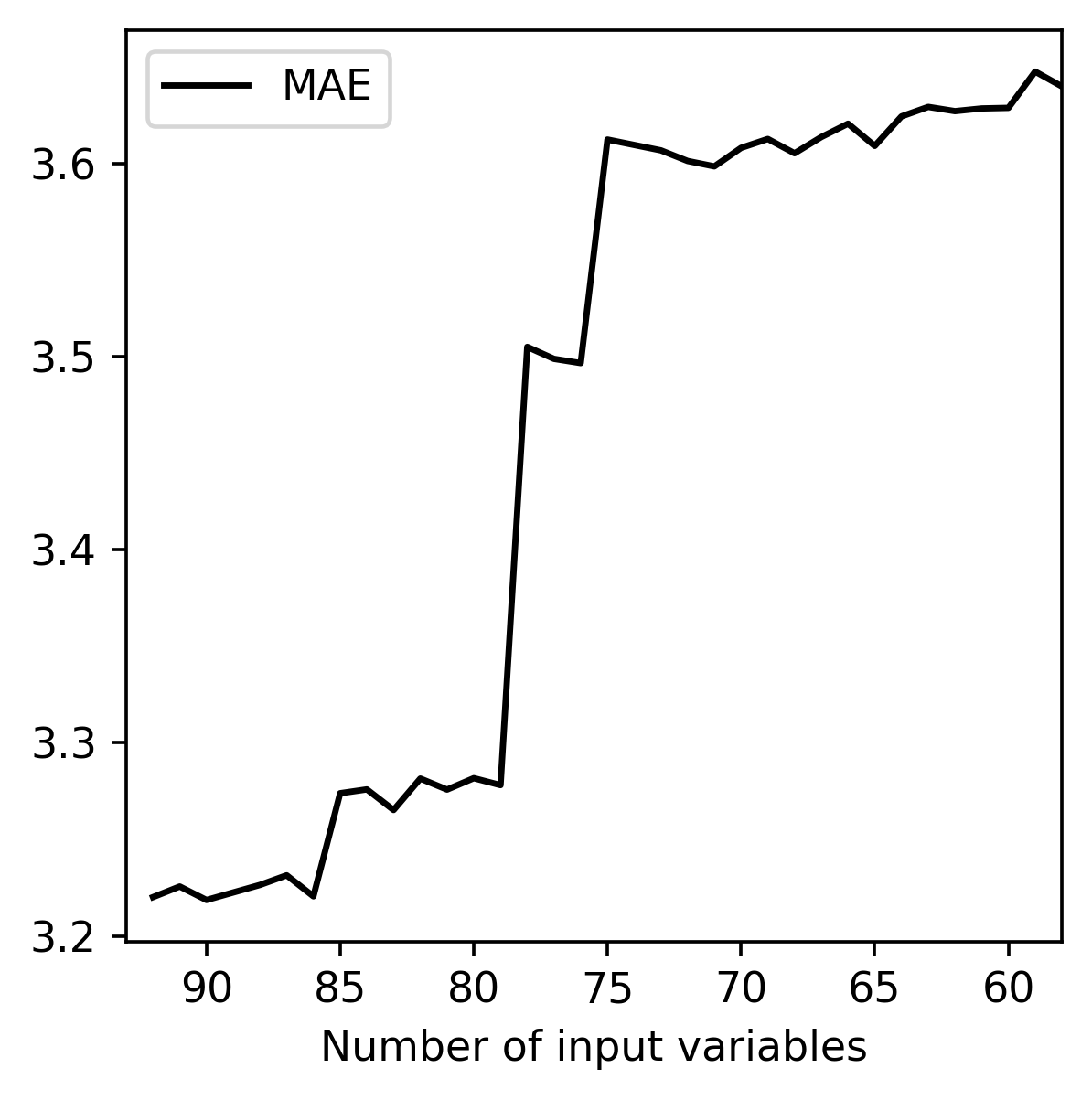   1. MAE | 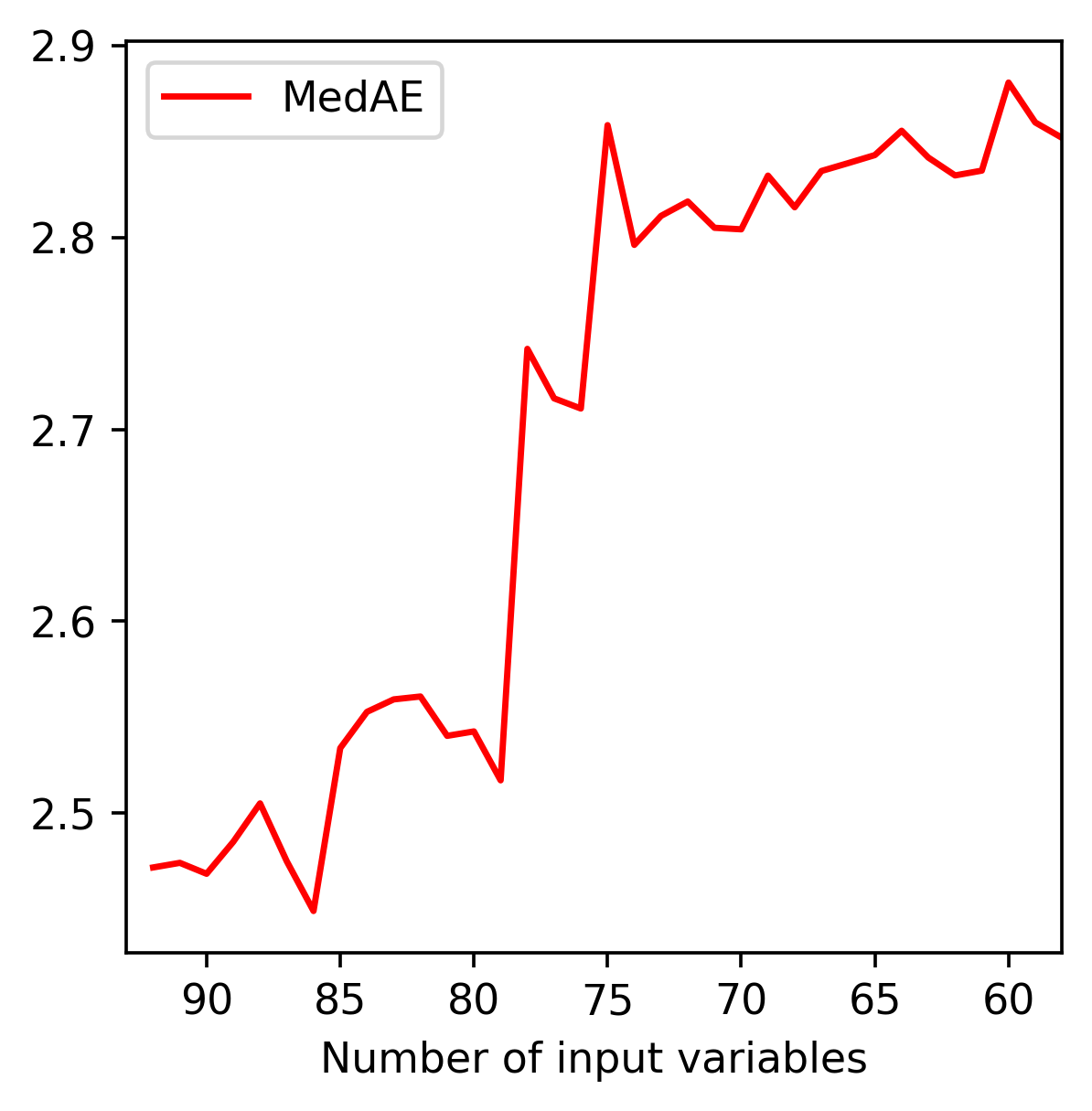   1. MedAE |
| --- | --- |
| 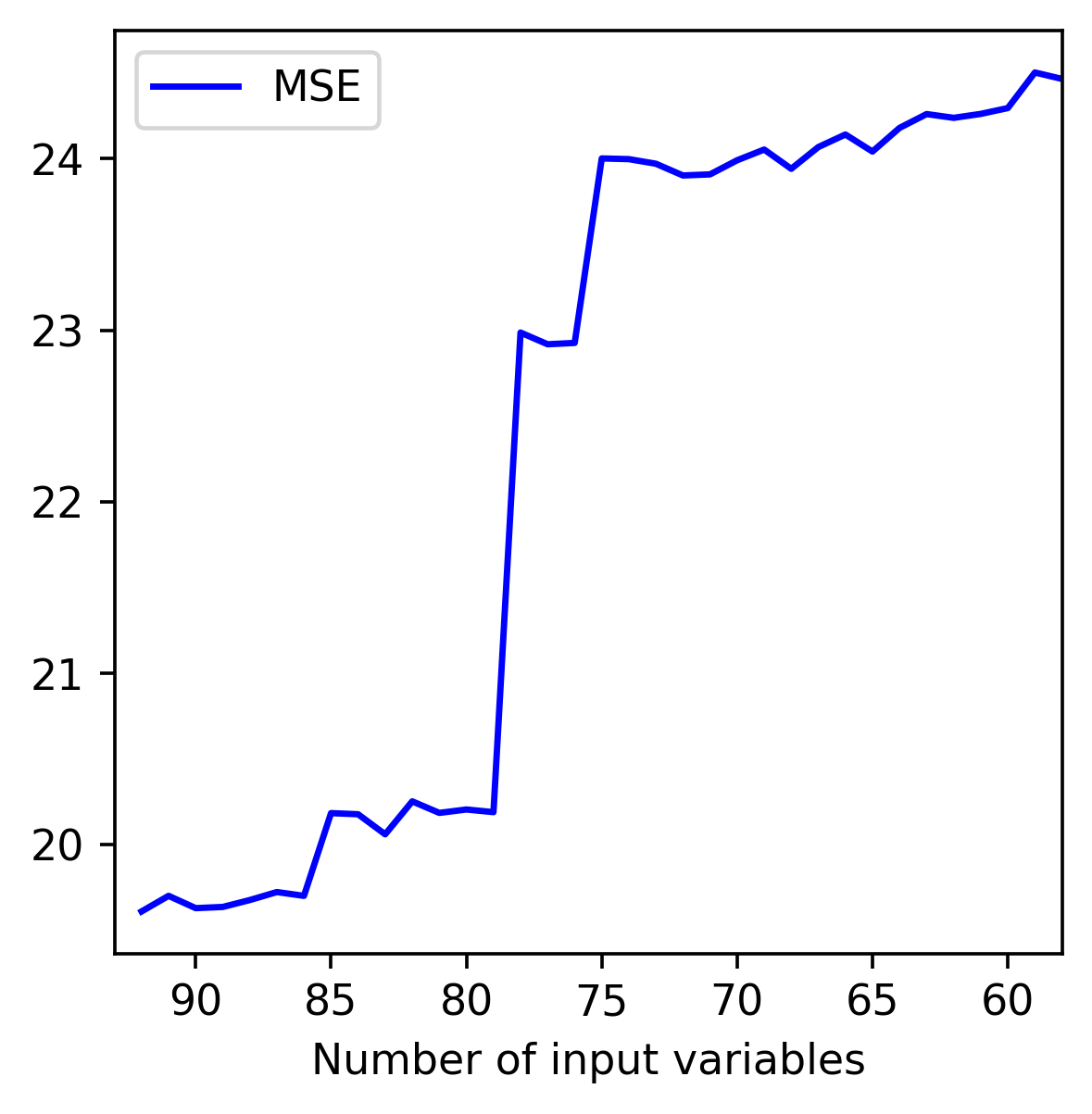   1. MSE | 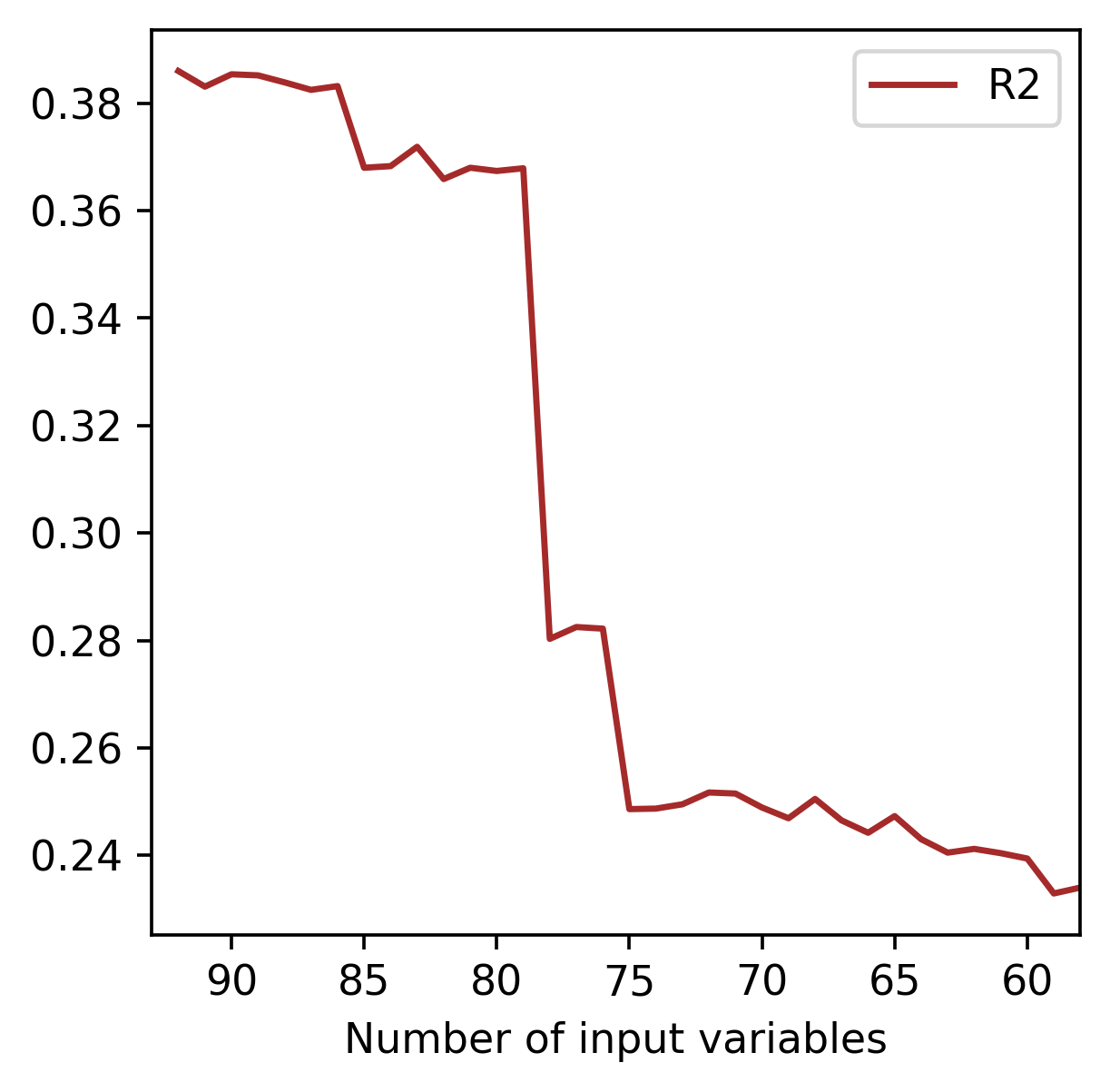   1. $R^{2}$ |
| 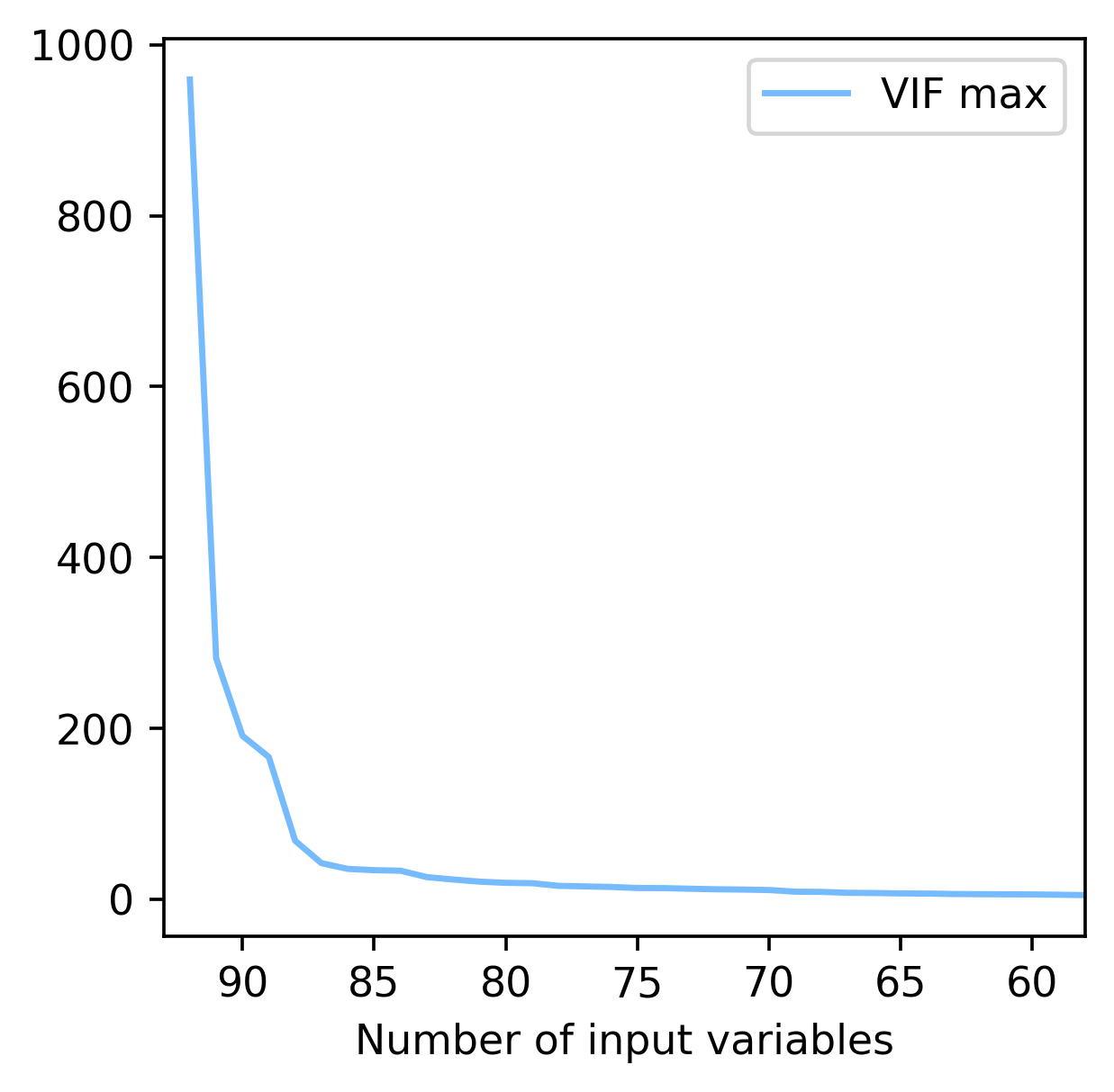   1. Max VIF value | 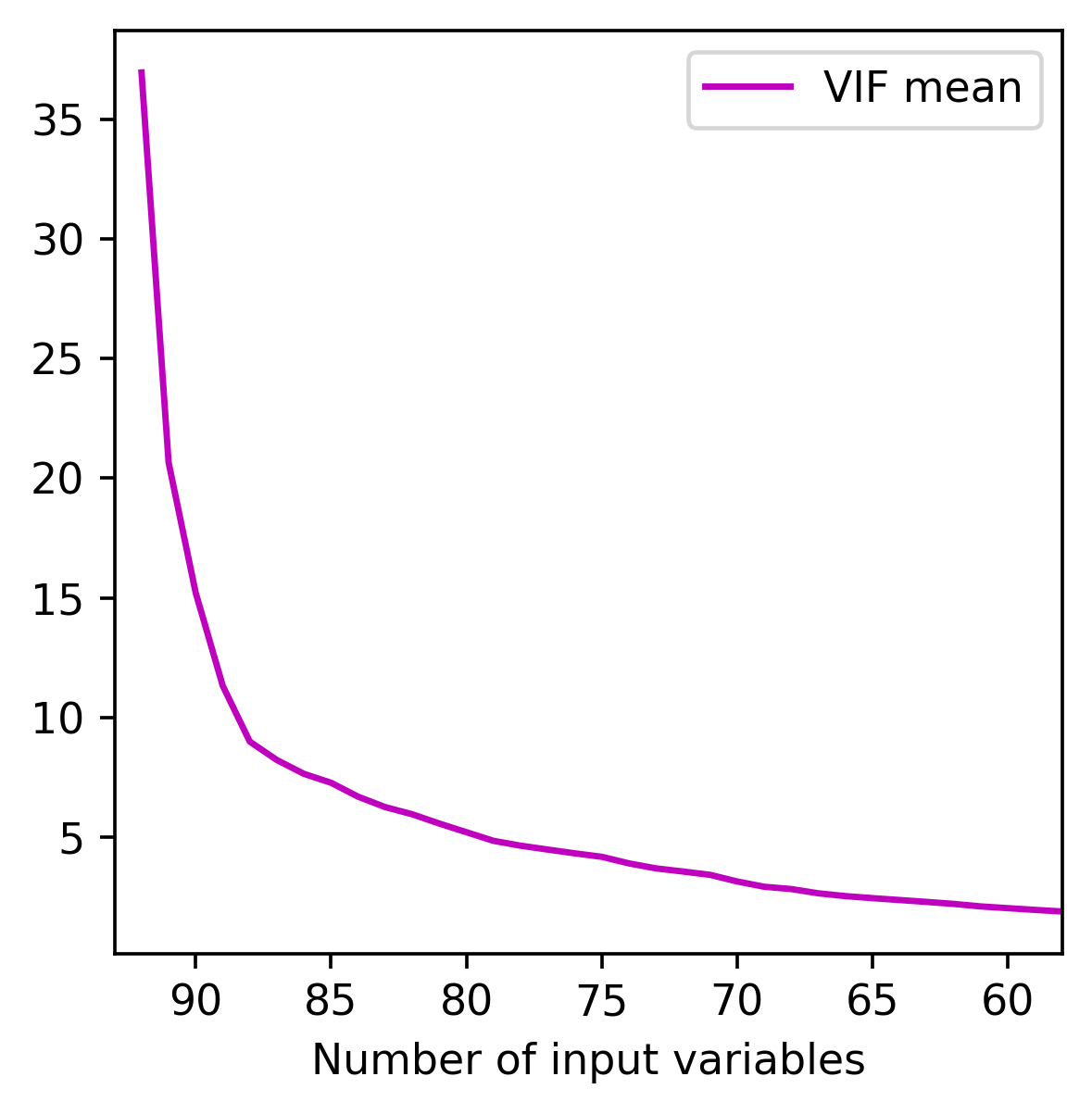   1. Mean VIF value |

Figure S3. CatBoost performance and degree of multicollinearity during the VIF-based RFE process

To further prove the reliability of the adopted machine learning models, we also compare all the models on the original dataset (93 input features, the dependent variable is still filled with KNN for missing values, N=25586) using 5-fold cross-validation, and it can be found that CatBoost is still the optimal model. Most machine learning models are better than traditional linear regression models.

Table S3. CV compare results

| Model | MAE | MSE | MedAE | R2 |  |
| --- | --- | --- | --- | --- | --- |
| XGBoost | 3.543(0.016) | 22.657(0.22) | 2.724(0.029) | 0.32(0.012) |  |
| GBDT | 3.533(0.026) | 21.158(0.286) | 2.85(0.033) | 0.365(0.014) |  |
| RF | 3.415(0.028) | 21.214(0.342) | 2.664(0.043) | 0.363(0.014) | |
| LightGBM | 3.475(0.027) | 21.046(0.319) | 2.748(0.029) | 0.368(0.012) | |
| CatBoost | **3.321**(0.031) | **20.842**(0.319) | **2.51**(0.042) | **0.374**(0.013) |  |
| LR | 3.666(0.018) | 22.366(0.241) | 2.961(0.038) | 0.328(0.017) |  |
